# Analysis of Polybrominated Diphenyl Ethers and Lipid Composition in Human Breast Milk and Their Correlation with Infant Neurodevelopment

**DOI:** 10.3390/ijerph182111501

**Published:** 2021-11-01

**Authors:** Ming-Hsien Tsai, How-Ran Chao, Wen-Li Hsu, Ching-Chung Tsai, Chu-Wen Lin, Chu-Huang Chen

**Affiliations:** 1 Department of Child Care, College of Humanities and Social Sciences, National Pingtung University of Science and Technology, Pingtung 91201, Taiwan; alantsai@mail.npust.edu.tw (M.-H.T.); cwlin@mail.npust.edu.tw (C.-W.L.); 2 Regenerative Medicine and Cell Therapy Research Center, Kaohsiung Medical University, Kaohsiung 80708, Taiwan; 1098129@kmuh.org.tw; 3 Emerging Compounds Research Center, General Research Service Center, National Pingtung University of Science and Technology, Pingtung 91201, Taiwan; 4 Department of Environmental Science and Engineering, College of Engineering, National Pingtung University of Science and Technology, Pingtung 91201, Taiwan; 5 Institute of Food Safety Management, College of Agriculture, National Pingtung University of Science and Technology, Pingtung 91201, Taiwan; 6 Department of Dermatology, Kaohsiung Municipal Ta-Tung Hospital, Kaohsiung Medical University Hospital, Kaohsiung Medical University, Kaohsiung 80145, Taiwan; 7 Department of Pediatrics, E-Da Hospital, Kaohsiung 82445, Taiwan; ed102514@edah.org.tw; 8 School of Medicine, College of Medicine, I-Shou University, Kaohsiung 82445, Taiwan; 9 Vascular and Medicinal Research, Texas Heart Institute, Houston, TX 77030, USA; cchen@texasheart.org; 10 New York Heart Research Foundation, Mineola, NY 11501, USA; 11 Institute for Biomedical Sciences, Shinshu University, Nagano 390-8621, Japan

**Keywords:** infant neurodevelopment, polybrominated diphenyl ethers, lipid, fatty acid, lipidomics

## Abstract

Breastfeeding is recommended over formula feeding, but human breast milk (HBM) composition varies and can be affected by food additives. Whether flame-retardant polybrominated diphenyl ethers (PBDEs) found in HBM interact with lipid components of HBM to impede infant neurodevelopment is a critical public health issue. Using lipidomic analysis, we examined the association of PBDEs in HBM and HBM lipid components with infant neurodevelopment. HBM samples (n = 100) were collected at the beginning stage of breastfeeding and analyzed for 30 PBDE congeners as well as a group of lipid components by using high-resolution gas chromatography, mass spectrometry, and liquid chromatography time-of-flight mass spectrometry. Infants were examined at 8 to 12 months of age by using the Bayley-III to assess neurodevelopment. A total of seven PBDEs, 35 lipids, and 27 fatty acids in HBM showed significant associations with Bayley-III scores. Multivariate analysis confirmed that these candidate PBDEs and lipid components were significant predictors of infant neurodevelopment. Eicosapentaenoic acid and docosapentaenoic acid in HBM showed no association with infant neurodevelopment in the general Taiwanese population. While certain PBDEs may play a role, our findings indicate that the lipid components of HBM are directly important for infant neurodevelopment.

## 1. Introduction

Polybrominated diphenyl ethers (PBDEs) are used as flame-retardant additives in a variety of products, such as electronic equipment and plastics [1], and are sold commercially as technical mixtures of penta-, octa-, and deca-BDEs [2]. However, the production and use of these mixtures have been banned or limited in some countries because of persistence and resistance to degradation [3]. Having been labeled as persistent organic pollutants, PBDEs continue to be ubiquitously present in the environment, even after limitations on their use were implemented. At the present time, PBDEs can be detected globally in various environments [4,5,6] and in the lipid-rich tissues of animals and humans [7,8,9]. The common presence of PBDEs has raised public concern because of the negative effects PBDEs have on the environment and humans. Evidence from animal and human studies has indicated that PBDEs may impair neurodevelopment, induce thyroid hormone disruption, affect reproductive and immune systems, and potentially cause cancer [10].

A substantial amount of evidence has linked PBDE exposure with developmental delay in humans, particularly in infants and children because PBDE concentrations are higher in these age groups than in adults [11]. Chao et al. [12] and Gascon et al. [13] reported that postnatal exposure to PBDEs in breast milk, particularly BDE-209, could potentially delay neurologic and mental development in nursing infants at ages 8 to 18 months. Furthermore, prenatal exposure to PBDEs may also affect cognition and delay the adaptive behavioral development of children [14]. For example, BDE-47 in serum was found to be associated with poorer cognitive abilities, as demonstrated by a lower intelligence quotient (IQ) observed in 5- and 8-year-old children who were exposed prenatally [15].

Human breast milk (HBM) lipids are mainly composed of triglycerides, which are esters derived from three fatty acids and glycerol. Polyunsaturated fatty acids (PUFAs) constitute 0.8% to 26% of triglycerides in HBM and are considered one of the most crucial lipids required for infant development [16]. HBM contains substantial amounts of PUFAs, such as the omega-3 docosahexaenoic acid (DHA, omega-3), eicosapentaenoic acid (EPA, omega-3), and arachidonic acid (ARA, omega-6), which have been suggested to play important roles in infant neurodevelopment, immune and tissue functions, growth, and health [17]. Levels of DHA and ARA, along with levels of other PUFAs, increase rapidly in the infant brain during the late stages of pregnancy and during the early stages of infancy [18]. DHA in the brain is involved in neuronal signaling, and ARA mediates neuronal firing, signaling, and long-term potentiation [19]. Low levels of DHA in the brain were found to be associated with poorer cognitive development [18]. In addition to lipids in HBM, lipids in other tissues have also been correlated with infant development. For instance, Brei et al. [20] reported that children exposed to higher concentrations of DHA, ARA, and EPA in cord blood showed more advanced neurodevelopment in terms of mirror movements, although the differences were not statistically significant. While we are continuously learning more about the role of lipids—especially PUFAs—in infant neurodevelopment, lipids represent a large group of macronutrients, constituting many components that remain unstudied. Thus, identifying the contributions of lesser-known lipids in HBM on infant neurodevelopment is an important area of study. Here, we analyzed HBM for PBDEs and lipids and studied the correlation of those specific PBDEs and lipids with infant neurodevelopment.

## 2. Materials and Methods

### 2.1. Study Participants

Study participants were pairs of healthy mothers and infants recruited at local hospitals in southern Taiwan between April 2007 and March 2011. The study protocol was reviewed and approved by the institutional review boards of the Human Ethical Committees of Pingtung Christian Hospital (PCH) in 2007 (NO: IRB021). Pregnant women were randomly recruited from the obstetric clinics of local hospitals during routine health check-ups as described previously [21,22]. More than 500 pregnant women were recruited, and 358 participants enrolled. Of the 358 pregnant participants, 265 completely answered the detailed questionnaire. A group of 145 participants voluntarily donated HBM. Of these, seven women were excluded due to insufficient HBM samples. Therefore, a total of 138 mothers from this cohort provided sufficient breast milk (>90 mL) for the chemical analysis of PBDEs. After delivery, the nursing infants of the 138 mothers were enrolled for a follow-up evaluation at the ages of 8 to 12 months. The participating mothers were contacted by telephone and were asked to bring their infants to the Department of Pediatrics at PCH for an assessment of infant development. More than 95% of the infants in this cohort participated in the follow-up program and were evaluated by pediatricians. Ultimately, 100 of these mother-infant pairs were included in the study on the basis of exclusive or partial breastfeeding during the first six months of lactation. A detailed flowchart of participant recruitment, enrollment, and inclusion is shown in Figure S1. HBM was collected within one month after delivery. Birth outcomes including gestational age, birth weight, birth length, and head circumference were recorded at birth by the pediatricians. Lipid composition in the breast milk of the 100 mothers were determined by performing additional lipidomics analysis.

### 2.2. Chemical Analysis of PBDEs

PBDEs in the HBM samples were analyzed as described previously [12,22]. Briefly, 30 PBDE congeners including BDE-7, -15, -17, -28, -47, -49, -66, -71, -77, -85, -99, -100, -119, -126, -138, -139, -140, -153, -154, -156, -183, -184, -191, -196, -197, -203, -206, -207, -208, and -209 were analyzed by using high-resolution gas chromatography with a high-resolution mass spectrometer (HRGC/HRMS). HBM was analyzed with eight internal standards, including ^13^C_12_-labeled BDE-28, -47, -99, -153, -183, -197, -207, and -209, and it was extracted by means of sonication with a mixture of *n*-hexane and acetone. The extract was then centrifuged. This procedure was repeated at least three times. After sample extraction, the lipid content of the HBM was determined with the gravimetric method. The extract was concentrated and dissolved in *n*-hexane and treated with concentrated sulfuric acid for the cleanup procedure by passage through a multi-column system. The eluate was concentrated to near-dryness, redissolved in *n*-hexane, and then transferred to a vial. The final eluate was analyzed with HRGC/HRMS (Hewlett-Packard GC 6970/Micromass Autospec Ultima). Quantification was performed with internal/external standard mixtures prepared by using the isotope dilution method. The eight ^13^C_12_-labeled PBDE internal standards were added to the HBM before extraction to ensure recovery during the process of chemical analysis. The blank tests including solvent and glassware blanks were regularly checked to ensure quality control during experiments. Limits of detection (LODs) were predetermined so the signal-to-noise ratios for both ions including the quantifier and qualifier of a specific congener would be greater than three. The method detection limits (MDLs) of breast milk PBDEs were measured as 0.980 to 17.400 pg/g lipid for BDE-7 to BDE-208 and 115.00 pg/g lipid for BDE-209 only. For measurements below the MDLs, PBDE concentrations were recognized as half of the MDLs.

### 2.3. Chemical Analysis of Lipids

**Figure 1:**
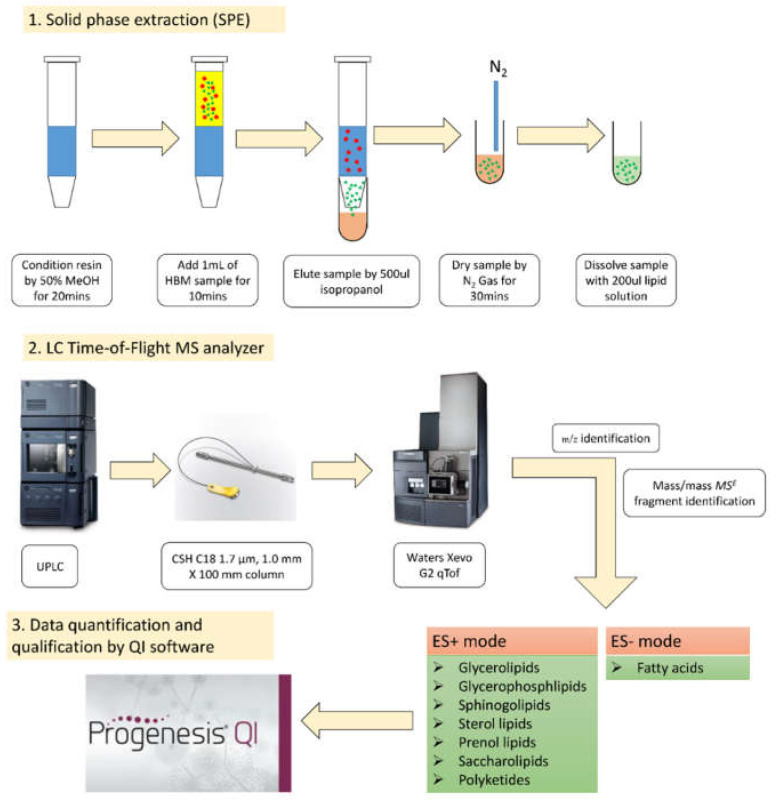
Steps of lipidomics analyses.

Figure 1 shows a schematic diagram of the lipidomic analyses. Lipids were extracted from HBM (n = 100) by using the Oasis^®^ HLB solid-phase extraction (SPE) method. Briefly, the SPE column resin was conditioned with 50% methanol. The HBM sample (1 mL) was then added into the SPE column and incubated. The sample was eluted with isopropanol alcohol and dried with nitrogen gas. Last, the sample was dissolved in lipid solution with a mixture of isopropanol/acetonitrile/water (2:1:1). Lipids were quantified with liquid chromatography data-independent, parallel fragmentation in mass spectrometry (LC/MS^E^). Briefly, lipids were chromatographically separated by using an ACQUITY ultra performance liquid chromatography separation system with a CSH™ C_18_ column. Mass spectrometry analysis was performed with a Xevo G2 quadrupole time-of-flight mass spectrometer (qTof) equipped with an electrospray ionization probe interface, with 3 kV for positive mode (ES+) and 2 kV for negative mode (ES−). The mass spectrometer was operated in the data-independent collection mode (MS^E^). Parallel ion fragmentation was programmed to switch between low (4 eV) and high (35–55 eV) energies in the collision cell, and data were collected from 200 to 1600 *m*/*z*, utilizing leucine (*m*/*z* 556.2771 for ES+ and 554.2704 for ES−) as the separate data channel lock mass calibrant. Mass spectrum data were imported, processed, and identified by using the LIPID MAPS Structure Database (LMSD) 11 July 2019, updated with Nonlinear Progenesis QI software. The normalized abundance and raw abundance of the identified compounds were exported from QI software and prepared for further correlation analysis and univariate and multivariate analyses.

### 2.4. Neurodevelopmental Test

The neurodevelopment of infants was reviewed and assessed by infant psychometrists. The Bayley Scales of Infant and Toddler Development, Third Edition (Bayley-III), was used to examine neurologic and neurobehavioral development in infants. The Bayley-III score has five domains, including cognitive, language, and motor scales that were assessed by the infant psychometrist. The social–emotional and adaptive behavior scales were assessed by using two parent-report questionnaires that were answered by the parents [12,23]. The Bayley-III composite scores provided developmental quotients, including raw scores and chronological age, and generated continuous outcome scores for the cognitive, language, motor, social–emotional, and adaptive behavior scales.

### 2.5. Statistical Analysis

To investigate the associations of PBDEs, lipids, and/or fatty acids with the five domains of Bayley-III scores, we analyzed descriptive statistics and Spearman correlation and performed univariate and multivariate analyses. The mean, standard deviation, median, range, and 95% confidence interval of the mean (95% CI) were used to evaluate the statistical dispersion of PBDEs, lipids, and fatty acids. All statistical analyses, including the generalized estimating equation (GEE) models, were conducted by using Statistical Product and Service Solutions (SPSS) V13 and Statistical Analysis System (SAS). Quasilikelihood under the independence model criterion (QIC) was used as a goodness-of-fit statistic for the GEE models. The beta (β) estimates, 95% confidential intervals, and p-values were used to evaluate the effects under multivariate adjustment. Redundancy analyses (RDA) performed with the XLSTAT Microsoft Excel extension package (Addinsoft, New York, NY, USA) were used to investigate the canonical correlation of PBDEs, lipids, and/or fatty acids with the five domains of the Bayley-III score. Box-and-whisker plots of PBDEs were drawn by GraphPad Prism 5.0 software (GraphPad Software, San Diego, CA, USA).

## 3. Results

### 3.1. Demographic Characteristics of Study Participant Pairs

Table 1 shows the descriptive statistics of the study participant pairs (n = 100), along with the demographic data of the mothers and newborns. For the mothers, we reported the mean maternal age, highest level of education, family income, smoking status during pregnancy, alcohol consumption during pregnancy, prepregnant body mass index (BMI), and average number of years lived in the Kaohsiung-Pingtung (Kaoping) area. For the newborns, we reported sex, number breastfed for six months, number breastfed for longer than six months, gestational age (weeks), birth weight, birth length, head circumference, indoor smoking exposure, and the Bayley-III developmental scores, including cognitive, language, motor, social–emotional, and adaptive behavior scales. 

### 3.2. Association between HBM PBDEs and Bayley-III Scores

In Table 2, a total of 30 PBDE compounds and ΣPBDEs are listed by order of bromine number (from 2 to 10), along with the N > LOD, MDL, mean ± standard deviation, median, and 95% CI. Box-and-whisker plots are shown in Figure S2. The coefficient *r* was used to assess the associations between PBDEs and the five domains of the Bayley-III scale (Table 3). BDE-206 (*r* = −0.189, *p =* 0.06) and BDE-209 (*r* = −0.218, *p =* 0.03) showed significant negative associations with the cognitive domain. BDE-140 (*r =* 0.175, *p =* 0.081) and BDE-203 (*r =* 0.216, *p =* 0.031) showed positive associations with the social–emotional domain. The predominant PBDEs were BDE-47 (1620 ± 8130 pg/g lipid), BDE-153 (1080 ± 1140 pg/g lipid), and BDE-209 (863 ± 1940 pg/g lipid). The mean concentration of Σ_30_PBDEs was 5800 ± 12800 pg/g lipid. Significant associations were observed between the cognitive domain of the Bayley-III scale and the language and motor domains, with corresponding Spearman correlation coefficients (*r*) of 0.474 and 0.345 (*p* < 0.001), respectively (Table 3). Furthermore, the language domain was significantly associated with the motor domain (*r =* 0.371, *p* < 0.001) and the adaptive behavior domain (*r* = 0.316, *p* < 0.001); the motor domain was significantly associated with the adaptive behavior domain (*r =* 0.255, *p* < 0.05); and the social–emotional domain was significantly associated with the adaptive behavior domain (*r* = 0.385, *p* < 0.001).

### 3.3. Association between HBM Lipids and Bayley-III Scores

Using mass spectrometry, we obtained a total of 292 signals identified in ES+ mode, which indicated the category of lipid (i.e., glycerolipids, glycerophospholipids, sphingolipids, sterol lipids, prenol lipids, saccharolipids, and polyketides). Among the 292 lipids, 34 were shown to be significantly associated with the five domains of the Bayley-III scale (Table 4). The mean ± standard deviation, median, and 95% CI are listed in Table 4. The lipids were represented in terms of retention time and mass-to-charge ratio (rt_*m*/*z*). One lipid, designated 14.16_579.5402, showed a significant positive association with the cognitive domain (*r* = 0.221, *p* = 0.027). Nine lipids, designated 5.24_524.3729 (*r* = −0.227, *p* = 0.023), 6.05_507.4033 (*r* = −0.239, *p* = 0.016), 7.80_535.4360 (*r* = −0.219, *p* = 0.029), 11.09_615.4976 (*r* = −0.205, *p* = 0.041), 13.54_617.5134 (*r* = −0.202, *p* = 0.044), 14.32_661.5383 (*r* = −0.246, *p* = 0.014), 14.78_689.5731 (*r* = −0.198, *p* = 0.048), 14.78_726.6541 (*r* = −0.257, *p* = 0.01), and 15.28_769.6350 (*r* = −0.207, *p* = 0.039), showed negative associations with the language domain. On the other hand, one lipid, designated 14.32_622.6100 (*r* = 0.214, *p* = 0.032), showed a significant positive association with the language domain. One lipid, designated 1.02_348.2749 (*r* = −0.207, *p* = 0.039), showed a significant negative association with motor domain. Eight lipids, designated 0.83_309.2057 (*r* = 0.203, *p* = 0.043), 1.02_367.1932 (*r* = 0.261, *p* = 0.009), 1.02_369.2070 (*r* = 0.240, *p* = 0.016), 1.05_195.1378 (*r* = 0.197, *p* = 0.05), 1.05_311.2221 (*r* = 0.203, *p* = 0.042), 1.05_349.1826 (*r* = 0.232, *p* = 0.02), 1.09_293.2119 (*r* = 0.211, *p* = 0.035), and 9.11_537.5353 (*r* = 0.237, *p* = 0.017), showed significant positive associations with the social–emotional domain, whereas no lipids were found to have significant negative associations with the social–emotional domain. Last, seven lipids, designated 0.93_353.2246 (*r* = 0.217, *p* = 0.03), 1.02_335.2180 (*r* = 0.213, *p =* 0.033), 1.02_367.1932 (*r* = 0.232, *p* = 0.02), 1.05_349.1826 (*r* = 0.213, *p* = 0.034), 1.05_351.1958 (*r* = 0.247, *p* = 0.013), 1.09_330.2644 (*r* = 0.213, *p* = 0.033), and 16.52_902.8188 (*r* = 0.206, *p* = 0.04), showed significant positive associations with the adaptive behavior domain, whereas two lipids, designated 5.24_524.3729 (*r* = −0.205, *p* = 0.041) and 15.87_771.7168 (*r* = −0.203, *p* = 0.043), showed significant negative associations with the adaptive behavior domain. The average normalized abundance of the lipids (abundance mean ± standard deviation) was calculated and is shown in Table 4.

### 3.4. Association between HBM Fatty Acids and Bayley-III Scores

Using mass spectrometry, we obtained a total of 109 mass spectrometry signals identified in ES-mode for fatty acids, including EPA and DHA (Table 4 and Table S1). The fatty acids were presented in terms of rt_*m*/*z*. No fatty acids showed any significant associations with the cognitive domain. One fatty acid, designated 1.05_621.4340 (*r* = 0.201, *p* = 0.045), showed a significant positive association with the language domain. Another fatty acid, designated 1.52_249.1907 (*r* = 0.201, *p* = 0.045), showed a significant positive association with the motor domain. Eight fatty acids, designated 0.93_273.1837 (*r* = 0.254, *p* = 0.011), 0.93_323.1899 (*r* = 0.227, *p* = 0.023), 0.93_407.2098 (*r* = 0.207, *p* = 0.039), 0.98_325.2054 (*r* = 0.244, *p* = 0.015), 0.98_327.2215 (*r* = 0.259, *p* = 0.009), 0.98_329.2375 (*r* = 0.209, *p* = 0.037), 1.02_349.2050 (*r* = 0.209, *p* = 0.037), and 1.09_313.2398 (*r* = 0.216, *p* = 0.031), showed significant positive associations with the social–emotional domain. On the other hand, three fatty acids, designated 1.48_199.1758 (*r* = −0.243, *p* = 0.015), 2.72_255.2380 (*r* = −0.251, *p* = 0.012), and 2.79_537.4898 (*r* = −0.214, *p* = 0.033), showed significant negative associations with the social–emotional domain. Last, 11 fatty acids, designated 1.02_329.2911 (*r* = 0.198, *p* = 0.049), 1.02_349.2050 (*r* = 0.202, *p* = 0.044), 1.02_353.2364 (*r* = 0.197, *p* = 0.05), 1.05_309.2112 (*r* = 0.262, *p* = 0.009), 1.05_311.2272 (*r* = 0.282, *p* = 0.005), 1.05_311.2791 (*r* = 0.293, *p* = 0.003), 1.05_621.4340 (*r* = 0.297, *p =* 0.003), 1.09_313.2398 (*r* = 0.289, *p* = 0.004), 1.09_335.2255 (*r* = 0.219, *p* = 0.029), 1.21_293.2166 (*r* = 0.204, *p* = 0.042), and 1.21_295.2321 (*r* = 0.212, *p* = 0.035), showed significant positive associations with the adaptive behavior domain. On the contrary, three fatty acids, designated 1.48_199.1758 (*r* = -0.233, *p* = 0.020), 1.98_227.2067 (*r* = −0.250, *p =* 0.012), and 2.72_255.2380 (*r* = −0.207, *p* = 0.039), showed significant negative associations with the adaptive behavior domain. Interestingly, no significant associations were identified between EPA (1.71_301.2218) or DHA (1.91_327.2374) in HBM and the five domains of the Bayley-III scale (Table 4).

### 3.5. RDA Map Analysis of Bayley-III Scores with HBM PBDEs, Lipids, and Fatty Acids

**Figure 2:**
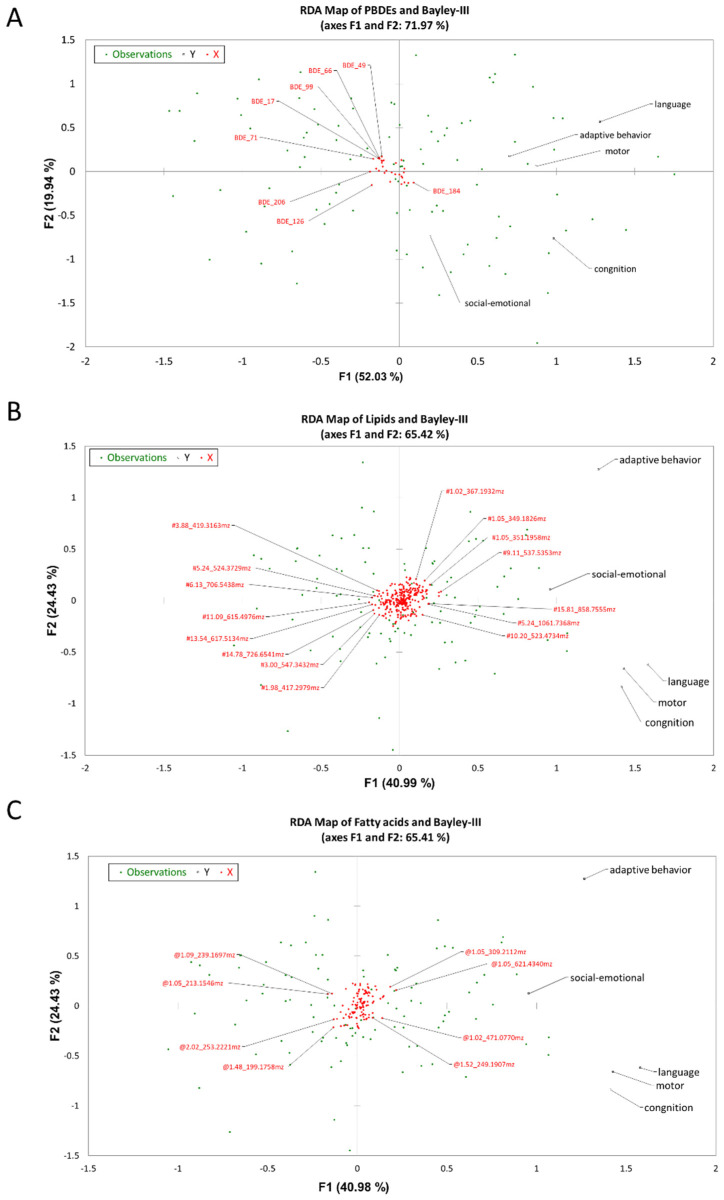
Redundancy analysis (RDA map) of PBDEs, lipids, fatty acids, and Bayley-III scores. (**A**) The canonical correlation between PBDEs and the five domains of Bayley-III. (**B**) The canonical correlation between lipids and the five domains of Bayley-III. (**C**) The canonical correlation between fatty acids and the five domains of Bayley-III.

RDA maps were used to extract and summarize the responses on Bayley-III scores. The PBDEs, lipids, and fatty acids were recognized as explained variables, and the responses were evaluated on a two-dimensional RDA biplot (Figure 2). The average abundances and *r* values are shown in Table 2 and Table 4. The RDA map of PBDEs and Bayley-III scores is shown in Figure 2A. Seven of the 30 PBDEs, which were BDE-17, -49, -66, -71, -99, -126, and -206, showed a strong negative correlation with the five domains of Bayley-III scores, indicating a highly negative impact. The RDA map of lipids and Bayley-III scores is shown in Figure 2B. Eight of the 292 lipids, designated 3.88_419.3163, 5.24_524.3729, 6.13_706.5438, 11.09_615.4976, 13.54_617.5143, 14.78_726.6541, 3.00_547.3432, and 1.98_417.2979, showed a strong negative correlation with the five domains of Bayley-III scores. Seven of the 292 lipids, designated 1.02_367.1932, 1.05_349.1826, 1.05, 351.1958, 9.11_537.5353, 5.81_858.7555, 5.24_1061.7368, and 10.20_523.4734, showed a strong positive correlation with the five domains of Bayley-III scores, suggesting a highly positive impact. Last, the RDA map of fatty acids and Bayley-III scores is shown in Figure 2C. Four of the 109 fatty acids, designated 1.09_239.1697, 1.05, 231.1546, 2.02_253.2221, and 1.48_199.1758, showed a strong negative correlation with the five domains of Bayley-III scores. In contrast, four of the 109 fatty acids, designated 1.05_309.2112, 1.05_621.4340, 1.02_471.0770, and 1.52_249.1907, showed a strong positive correlation with the five domains of Bayley-III scores.

### 3.6. Multivariate Analyses of HBM PBDEs, Lipids, Fatty Acids, and Bayley-III Scores

From 30 PBDEs, Σ30PBDEs, 292 lipids, and 109 fatty acids, we identified four PBDEs, 54 lipids, and 45 fatty acids that were significantly correlated with the five domains of Bayley-III scores in either correlation or RDA analyses. These PBDEs, lipids, and fatty acids were then utilized as independent variables in further multivariate analyses with generalized estimating equation (GEE) models in which we adjusted for confounding factors such as mother’s age, mother’s highest level of education, family income, mother’s smoking during pregnancy, prepregnant BMI, parity number of birth, living period in Kaoping area, newborn’s sex, gestational age (weeks), birth weight, birth length, and head circumference. The beta (β) estimates, 95% confidential intervals, and *p*-values are provided in Table 5. BDE-209, a lipid (15.47_868.7413) and a fatty acid (3.84_283.2688), showed significance in the cognitive model (QIC = 51.63). Two lipids (13.54_617.5134 and 15.47_868.7413) showed significance in the language model (QIC = 51.62). Two lipids (5.08_590.4762 and 14.32_661.5383) showed significance in the motor model (QIC = 50.08). Two lipids (5.08_590.4762 and 16.99_907.8456) and three fatty acids (0.93_171.1083, 1.02_353.2364, and 1.09_335.2255) showed significance in the social–emotional model (QIC = 48.41). Three lipids (1.02_348.2749, 1.05_173.1178, and 14.32_661.5383) showed significance in the adaptive behavior model (QIC = 50.68).

### 3.7. Information Related to Significant Lipids

Table 6 shows detailed information related to significant lipids including rt_*m*/*z* [ion], category [subclass], formula, common name, and lipid map link. In the adjusted multivariate assessment, seven lipids and four fatty acids were significant, which included the following: Two prenol lipids, two glycerophospholipids, two glycerol lipids, one sphingolipid, and four fatty acyls.

## 4. Discussion

In this study, we showed that PBDEs, lipids, and fatty acids in HBM samples play significant roles in infant neurodevelopment by demonstrating their positive or negative association with the five neurodevelopmental domains including cognitive, language, motor, social–emotional, and adaptive behavior domains. To our knowledge, this is the first report of the combined neurodevelopmental effects of PBDEs and lipid components in HBM. According to our findings, lipids in HBM have stronger effects on infant neurodevelopment than do PBDEs in HBM (or perhaps some toxicants with structural similarity to PBDEs). Our results support the breastfeeding policies of the Ministry of Health and Welfare in Taiwan.

PBDEs are widespread in the environment and enter into the human body through dietary (e.g., meat) and non-dietary sources (e.g., house dust). Because of the persistence and lipophilicity of PBDEs, they accumulate in adipose tissue and fatty cells around high-fat specimens such as breast milk. Recent reports have suggested that PBDEs negatively impact neurodevelopment in children. This is particularly critical for infants and young children who are believed to have a higher exposure to toxic chemicals than adults because of their larger chemical-to–body weight ratio (USEPA 2008). In a Spanish cohort of infants ages 12 to 18 months (n = 290), the Σ_7_PBDEs (including BDE-47, -99, -100, -153, -154, -183, and -209) were associated with decreased mental development scores, which was examined by using the Bayley Scales of Infant Development, Second Edition (BSID-II) [13]. However, an earlier study of American infants and children with a higher exposure to PBDEs suggested otherwise [24]. Herbstman et al. [24] reported that the congeners BDE-47, -99, -100, and -153 showed no significant effects on the mental development scores of infants at 12 months (n = 36), which was examined by using the BSID-II. Nonetheless, these congeners showed a significant negative association with psychomotor development [24]. A previous cohort study in Taiwan revealed a significantly higher odds ratio of low cognitive scores in infants (8–12 months) with higher levels of BDE-15, -99, -197, or Σ_11_PBDEs in cord blood (n = 36) [14]. They also showed that adaptive behavior was negatively correlated with BDE-28, -99, -154, -183, and Σ_11_PBDEs and that language, motor, and social–emotional development were not correlated with PBDEs [14]. We previously reported that specific PBDEs such as BDE-209 in HBM were negatively associated with cognitive development in infants at 8 to 12 months of age (n = 70) in Taiwan [12]. The present study also found that BDE-209 showed a significant negative correlation with the cognitive scale (Table 3). After performing multivariate analysis with confounders including maternal age, prepregnant BMI, infant gender, gestational age, and infant age at the time of testing, we found that BDE-209 was negatively associated with cognitive scores (B = −0.007, adjusted R = −0.224, *p* = 0.032), whereas BDE-196 was positively correlated with language scores (B = 0.096, adjusted R = 0.315, *p* = 0.002) [12]. We also found that BDE-206 was positively correlated with the adaptive behavior scale (Table S1). BDE-47, -99, -100, -153, and Σ_4_PBDEs were not significantly linked to any neurodevelopmental domains including motor, adaptive behavior, language, or social–emotional domains in infants at the age of 12 months old (n = 192) [25]. At the present time, few studies have investigated the association between PBDEs and infant neurodevelopment, and the findings have been inconsistent. Of note, these inconsistencies may be due to differences in the method used for assessing neurodevelopment (i.e., BSID-II or Bayley-III), sample size, measurement of PBDE congeners, or the population studied. Nevertheless, the findings support that PBDEs may cause neurodevelopmental decrements in infants. Most studies have focused on the neurodevelopmental effects of PBDEs in children between the ages of 2 and 12 years who were exposed in utero and during childhood. Among these studies, PBDE exposure was consistently associated with decreased cognitive functions, although not all results were statistically significant for certain ages [14,15,24,26]. For example, Chen et al. [26] reported that in children 1 to 3 years of age, BDE-47 was not significantly associated with mental or psychomotor development. However, in children 5 years of age, a decrease in full-scale IQ and an increase in hyperactivity were observed. Similarly, Braun et al. found that in children at 5 and 8 years of age, BDE-47 was associated with a lower IQ and more externalizing behaviors [15].

We also provide new evidence of a correlation between lipids in HBM and infant neurodevelopment. The lipids in HBM that have significant involvement in infant neurodevelopment are shown in Table 5. These lipids are classified into the following six following categories: Prenol lipids, glycerophospholipids, glycerolipids, sphingolipids, and fatty acyls. Below, we will discuss these lipids by category.

Prenol lipids. Two of the prenol lipids were identified as having a significant effect on infant neurodevelopment. Gibberellin A1 had a significant negative effect on the adaptive behavior development of infants (Table 5) and has been reported as a plant growth regulator [27]. On the other hand, 1α,3α,4β-p-menthane-3,8-diol had a positive effect on the regulation of adaptive behavior development (Table 5). One study investigated the modified structure of p-menthane-3,8-diol as a repellent against insects [28], but its biologic function in mammalian neurodevelopment has not been studied independently. 

Glycerophopholipids. PE(21:0/0:0) and PC(20:1(11Z)/22:2(13Z, 16Z)) are two glycerophospholipids that showed significant positive associations with social–emotional and motor development, but showed a negative association with language domain (Table 5 and Table 6). High-throughput lipidomics analysis has indicated that PE(21:0/0:0) may induce anti-cancer activity [29]. Decreased levels of PC(20:1(11Z)/22:2(13Z, 16Z)) have been associated with glaucomatous pathology [30]. Given that there is solid evidence showing that cancer or glaucoma is due to inflammatory responses, these glycerophospholipids may conceivably have the capacity to guard against inflammation [31,32].

Glycerolipids. Glycerolipids are composed of mono-, di-, and tri-substituted glycerols [33]. Here, we studied the diglycerides DG(18:2(9Z, 12Z)/18:2(9Z, 12Z)/0:0) and the triglyceride TG(18:3(9Z, 12Z, 15Z)/18:3(9Z, 12Z, 15Z)/20:0)[iso3]. The biologic functions of DG(18:2(9Z, 12Z)/18:2(9Z, 12Z)/0:0) remain unclear, but our results distinguished the unique negative effects of these diglycerides on infant social–emotional development (Table 5). Despite the close relationship between TG(18:3(9Z, 12Z, 15Z)/18:3(9Z, 12Z, 15Z)/20:0)[iso3] and alcoholic steatosis (Zhong et al. 2012), our results showed that TG(18:3(9Z, 12Z, 15Z)/18:3(9Z, 12Z, 15Z)/20:0)[iso3] deaccelerated language development (Table 5).

Sphingolipids. The analysis of lipid molecules with LC time-of-flight MS, sphingolipids were recognized as two molecularly distinct compositions: Ceramides and sphingomyelins. As shown in Table 5, most ceramides, such as PE-Cer(d14:1(4E)/20:0), negatively influenced the development of language. A previous study also showed the effect of Cer(d18:1/22:0) on ceramide-biosynthesis pathways in underweight and normal weight children [34]. While the sphingomyelin SM(d18:1/24:0) is involved in neurodegenerative diseases and is found at decreased levels in these patients [35], SM(d18:2/21:0) was negatively associated with the development of adaptive behavior. Understanding the biologic functions of these sphingolipids requires further investigation.

Fatty acyls. Fatty acyls play an important role in modulating infant neurodevelopment [36]. A well-known example is DHA, the most abundant omega-3 fatty acid in the brain, which promotes brain development and function [37]. Most fatty acyls, as shown in Table 3 and Table 5, tend to accelerate neurodevelopment in the areas of language, motor, social–emotional, and/or adaptive behavior. These fatty acyls include saturated fatty acids, capric acid, octadecapentaenoic acid, PGF2α, and α-licanic acid. Both capric acid and DPA have been shown to alleviate oxidative stress, inflammatory cytokine production, and gene expression related to oxidative stress [38,39]. Docosapentaenoic acid (DPA) has been shown to advance neurodevelopment [37]. While PGF2α has been shown to enhance neuronal cell death and lead to neurodegeneration [40], our results indicated that increased PGF2α shows a significant positive correlation with adaptive behavior. Unexpectedly, we found that palmitic acid, which is the major saturated fatty acid in human milk comprising 17% to 25% of the total fatty acids [41], was negatively associated with social–emotional and adaptive behavior development. Interestingly, palmitic acid, cell cycle G2/M arrest, and endoplasmic reticulum stress trigger neuron cell apoptosis [42]. Polyunsaturated fatty acid mayolene-18 was also associated with reduced motor development, but there is no clear evidence to explain how mayolene-18 affects infant neurodevelopment. Overall, most of the identified lipids in breast milk were positively correlated with infant neurodevelopment, although some lipids were negatively correlated with infant neurodevelopment.

This study had limitations. Breast milk components including lipids and fatty acids may vary daily with the mothers’ dietary intake, drinking water consumption, drug use, and physiologic and psychologic conditions. The breast milk samples were provided by the PBDE survey studies, designed for the surveillance of PBDEs in breast milk to explore the association of breast milk PBDEs with adverse health effects in pregnant women and their nursing infants [7,22,43,44,45,46]. PBDEs are lipophilic, accumulate in fatty tissues or adipose cells, and are resistant to physical, chemical, and biological degradation. The variation of PBDE levels in first-month breast milk is minor. Based on the design of the PBDE survey study, we did not require our study participants to provide breast milk after the first month. The varied composition of lipids and fatty acids in breast milk during the first month was not controlled in the present study.

Our study provided evidence of significant associations between PBDEs, lipids, and fatty acids and infant neurodevelopment. However, we acknowledge that our study has several limitations that should be addressed in future studies. First, although we were able to adjust for potential confounders, we cannot rule out the effects of unmeasured confounders that may affect infant neurodevelopment, such as diet, lifestyle, and social class of the mothers. Second, we did not separate the analysis between partially and exclusively breastfed infants. Partial or exclusive breastfeeding may have a different magnitude of effects on infant neurodevelopment. Third, we considered only infants with postnatal exposure to PBDEs through HBM. While lipids were already present in HBM and could enter the infant’s body via breastfeeding, PBDEs in formula milk and solid food were not studied. Last, we did not measure hydroxylated PBDE metabolites, which may have a greater effect on neurodevelopment because their toxicities are greater than those of parent PBDEs [47].

## 5. Conclusions

Environmental pollutants such as PBDEs have known negative effects on infant and child neurodevelopment, as shown by observations made in in vitro, in vivo, and epidemiologic studies. In contrast, nutrients in HBM such as lipids are known to be essential for infant development. However, few studies have shown the negative effects of lipids on neurodevelopment. This topic has recently emerged as an important area of study, especially in the fields of environmental health and pediatrics. Our study showed that certain PBDEs in HBM are associated with infant neurodevelopment, particularly in the cognitive and social–emotional domains. HBM lipid components, including lipids, showed significant associations with all the domains. Compared with HBM PBDEs, HBM lipids showed the greatest impact on infant neurodevelopment. Future research is warranted to investigate the mechanistic pathways by which lipids are associated with neurodevelopment. Furthermore, additional epidemiologic studies to investigate the association between lipids and infant and childhood neurodevelopment are needed to further substantiate our study findings.

## Figures and Tables

**Table 1:** Descriptive statistics of the study participant pairs and demographic data of the mothers and newborns (n = 100).

Variable	Mean ± SD	Range	n (%)
Mothers			
Age (y)	29.5 ± 4.64	17.0–40.0	
Highest level of education			
Below senior level in high school	-	-	5 (5%)
Senior level in high school	-	-	26 (26%)
Junior college	-	-	28 (28%)
University	-	-	35 (35%)
Graduate school	-	-	6 (6%)
Family income			
<300,000 NTD	-	-	14 (14%)
300,000–600,000 NTD	-	-	32 (32%)
600,000–1,000,000 NTD	-	-	39 (39%)
1,000,000–1,500,000 NTD	-	-	11 (11%)
>1,500,000 NTD	-	-	4 (4%)
Smoking during pregnancy, Yes	-	-	4 (4%)
Alcohol consumption during pregnancy, Yes	-	-	0 (0%)
Prepregnant BMI (kg/m^2^)	22.5 ± 4.1	15.4–34.9	
Parity (number)	1.9 ± 0.8	1.0–4.0	
Living period in Kaoping area (y)	22.2 ± 11.6	1.0–40.0	
Newborns			
Female	-	-	51 (51%)
Breastfed for 6 months, Yes	-		100 (100%)
Breastfed for more than 6 months, Yes	-	-	0 (0%)
Gestational age (weeks)	38.5 ± 1.4	32.0–44.0	
Birth weight (kg)	3.10 ± 0.36	2.30–4.12	
Birth length (cm)	49.1 ± 1.7	44.0–55.0	
Head circumference (cm)	33.5 ± 1.3	30.5–36.0	
Indoor smoking exposure, Yes	-	-	10 (10%)
Neurodevelopment			
Infants (Bayley-III)			
Cognitive scale (score)	104.0 ± 10.5	85.0–130.0	
Language scale (score)	102.0 ± 12.1	74.0–131.0	
Motor scale (score)	99.7 ± 9.8	79.0–130.0	
Social–emotional scale (score)	98.5 ± 18.2	55.0–140.0	
Adaptive behavior scale (score)	100.0 ± 14.3	62.0–133.0	

Abbreviations: BMI, body mass index; SD, standard deviation.

**Table 2:** Levels of 30 PBDE homologues in human breast milk of study participants from Kaohsiung and Pingtung areas of southern Taiwan (pg/g lipid) (n = 100).

PBDEs (Bromine Number)	N > LOD ^a^	MDL ^b^	Abundance (Mean ± SD)	Median	95% CI
BDE-7 (2 Br)	11	1.04	1.08 ± 0.74	0.90	0.93 ^c^–1.22 ^d^
BDE-15 (2 Br)	100	0.98	69.00 ± 73.00	46.00	54.50–83.50
BDE-17 (3 Br)	11	3.07	5.53 ± 6.63	3.97	4.22–6.85
BDE-28 (3 Br)	100	1.85	128.00 ± 440.00	65.20	40.50–215.00
BDE-47 (4 Br)	100	17.4	1620.00 ± 8130.00	535.00	2.74–3230.00
BDE-49 (4 Br)	98	3.87	44.70 ± 116.00	32.50	21.60–67.70
BDE-66 (4 Br)	96	3.45	23.60 ± 87.70	12.40	6.15–41.00
BDE-71 (4 Br)	10	4.33	4.26 ± 4.14	3.25	3.44–5.08
BDE-77 (4 Br)	30	1.28	1.31 ± 1.33	0.78	1.31–1.58
BDE-85 (5 Br)	78	1.37	24.80 ± 113.00	8.36	2.41–47.10
BDE-99 (5 Br)	100	10.0	391.00 ± 1960.00	145.00	547.00–800.00
BDE-100 (5 Br)	100	3.18	402.00 ± 1280.00	190.00	148.00–656.00
BDE-119 (5 Br)	48	1.95	18.90 ± 17.70	11.50	15.40–22.40
BDE-126 (5 Br)	20	0.996	2.27 ± 15.60	0.29	−0.83–5.36
BDE-138 (6 Br)	72	1.98	11.60 ± 28.20	6.11	5.95–17.10
BDE-139 (6 Br)	76	2.04	16.30 ± 38.30	10.10	8.65–23.80
BDE-140 (6 Br)	76	1.00	10.50 ± 14.60	7.61	7.57–13.40
BDE-153 (6 Br)	100	7.04	1080.00 ± 1140.00	851.00	851.00–1300.00
BDE-154 (6 Br)	100	1.04	112.00 ± 141.00	69.20	84.00–140.00
BDE-156 (6 Br)	0	1.02	0.51 ± 0.00	0.51	-
BDE-183 (7 Br)	98	4.88	206.00 ± 602.00	112.00	86.60–326.00
BDE-184 (7 Br)	30	3.01	8.12 ± 7.11	6.09	6.71–9.53
BDE-191 (7 Br)	14	4.11	6.20 ± 2.73	2.06	5.66–6.74
BDE-196 (8 Br)	96	6.06	39.00 ± 44.50	26.1	30.10–47.80
BDE-197 (8 Br)	100	2.94	262.00 ± 293.00	171.00	204.00–320.00
BDE-203 (8 Br)	99	7.23	71.20 ± 82.30	45.50	54.90–87.50
BDE-206 (9 Br)	76	1.74	91.30 ± 239.00	46.00	43.80–139.00
BDE-207 (9 Br)	100	1.53	212.00 ± 288.00	116.00	154.00–269.00
BDE-208 (9 Br)	96	1.11	83.30 ± 143.00	44.10	54.80–112.00
BDE-209 (10 Br)	98	115.00	863.00 ± 1940.00	341.00	479.00–1250.00
Σ_30_PBDEs	-	-	5800.00 ± 12,800.00	3400.00	3270.00–8340.00

Abbreviations: BDE, brominated diphenyl ethers; LOD, limit of detection; MDL, method detection limit; PBDE, polybrominated diphenyl ethers. ^a^ Number of samples below the LOD. ^b^ Method detection limit. ^c^ Lower bound. ^d^ Upper bound.

**Table 3:** Descriptive statistics and Spearman correlation of PBDEs with the five domains of the Bayley-III score (n = 100).

Variables	Cognitive (*r*)	Language (*r*)	Motor (*r*)	Social–Emotional (*r*)	Adaptive Behavior (*r*)
Bayley-III score					
Cognitive	1	0.474 ***	0.345 ***	0.149	0.078
Language	0.474 ***	1	0.371 ***	0.134	0.316 **
Motor	0.345 ***	0.371 ***	1	0.053	0.255 *
Social–emotional	0.149	0.134	0.053	1	0.385 ***
Adaptive behavior	0.078	0.316 **	0.255 *	0.385 ***	1
PBDEs (pg/g lipid)					
BDE-17 (3 Br)	−0.058	0.061	0.048	−0.019	−0.006
BDE-49 (4 Br)	−0.039	0.089	0.061	−0.040	−0.022
BDE-66 (4 Br)	−0.025	0.040	0.016	−0.004	−0.018
BDE-71 (4 Br)	−0.003	0.063	−0.030	0.014	−0.022
BDE-99 (5 Br)	−0.109	0.077	−0.139	−0.083	−0.045
BDE-126 (5 Br)	−0.069	−0.135	−0.047	−0.046	−0.081
BDE-140 (6 Br)	0.111	0.015	0.095	0.175 †	0.022
BDE-184 (7 Br)	0.152	0.056	0.076	0.107	−0.023
BDE-203 (8 Br)	−0.077	−0.051	−0.005	0.216 *	0.029
BDE-206 (9 Br)	−0.189 †	0.026	0.055	0.024	−0.008
BDE-209 (10 Br)	−0.218 *	0.150	0.043	−0.056	0.028
Σ_30_ PBDEs	−0.135	−0.087	−0.028	−0.075	−0.0360

Abbreviations: BDE, brominated diphenyl ethers; PBDE, polybrominated diphenyl ethers. † *p* < 0.1, * *p*<0.05, ** *p* < 0.01, *** *p* < 0.001.

**Table 4:** Descriptive statistics and Spearman correlation coefficient of significant lipids vs. the five domains of the Bayley-III score (n = 100).

Variables	Abundance (Mean ± SD)	Median	95% CI	Cognitive (*r*)	Language (*r*)	Motor (*r*)	Social–Emotional (*r*)	Adaptive Behavior (*r*)
Lipids (rt_*m*/*z*)								
0.83_309.2057	1366.66 ± 1013.64	1202.45	1165.53 ^c^–1567.79 ^d^	−0.004	0.010	−0.146	0.203 *	0.138
0.93_353.2246	2426.53 ± 1583.85	2177.67	2112.26–2740.80	0.017	0.016	−0.121	0.157	0.213 *
1.02_335.2180	2500.73 ± 1680.54	2125.61	2167.27–2834.18	0.103	0.083	−0.075	0.064	0.213 *
1.02_348.2749	3723.28 ± 3159.94	3034.29	3096.28–4350.28	−0.024	−0.029	−0.207 *	0.196 †	0.095
1.02_367.1932	1662.83 ± 908.85	1533.17	1482.49–1843.16	−0.023	0.011	−0.082	0.261 **	0.232 *
1.02_369.2070	2453.69 ± 1599.47	2230.90	2136.31–2771.05	−0.034	−0.076	−0.099	0.240 *	0.168 †
1.05_173.1178	661.22 ± 429.86	577.72	575.93–746.51	0.02	0.019	−0.148	0.193 †	0.150
1.05_195.1378	692.75 ± 446.62	601.81	604.13–781.37	0.046	0.054	−0.125	0.197 *	0.158
1.05_311.2221	2140.96 ± 1291.01	1772.66	1884.79–2397.12	−0.023	0.007	−0.152	0.203 *	0.145
1.05_349.1826	2099.01 ± 1031.09	1891.40	1894.42–2303.60	0.123	0.081	−0.039	0.232 *	0.213 *
1.05_351.1958	6779.36 ± 4081.45	5925.57	5969.51–7589.21	0.146	0.103	−0.018	0.164	0.247 *
1.09_293.2119	2669.1 ± 1541.38	2240.20	2363.26–2974.95	0.018	0.056	−0.123	0.211 *	0.160
1.09_330.2644	4647.12 ± 3779.65	3644.02	3897.15–5397.08	0.090	0.121	−0.096	0.149	0.213 *
1.98_417.2979	1758.6 ± 548.34	1750.95	1649.80–1867.40	−0.03	−0.170 †	0.057	−0.189 †	−0.176 †
5.08_590.4762	5523.24 ± 2915.93	4729.03	4944.65–6101.82	0.012	0.012	−0.048	0.196 †	0.076
5.24_524.3729	79.82 ± 44.95	73.78	70.90–88.74	−0.131	−0.227 *	−0.042	−0.009	−0.205 *
6.05_507.4033	1730.66 ± 846.35	1643.39	1562.73–1898.59	−0.129	−0.239 *	−0.049	−0.007	−0.165
7.80_535.4360	3103.57 ± 871.52	3188.12	2930.64–3276.50	−0.045	−0.219 *	−0.016	−0.012	−0.193 †
9.11_537.5353	1320.85 ± 2415.3	367.25	841.60–1800.10	0.116	0.138	−0.040	0.237 *	0.056
10.16_564.4132	22,252.96 ± 6920.35	21,384.68	20,879.81–23,626.10	0.040	0.059	0.166 †	−0.042	−0.024
11.09_615.4976	8052.28 ± 2152.2	8109.02	7625.24–8479.32	−0.097	−0.205 *	0.010	−0.110	−0.159
11.90_565.5672	3527.17 ± 6739.33	810.42	2189.94–4864.40	0.105	0.111	−0.023	0.189 †	0.065
13.54_617.5134	3498.78 ± 819.03	3424.17	3336.27–3661.30	−0.069	−0.202 *	0.046	−0.094	−0.100
14.16_579.5402	3000.3 ± 1960.65	2524.79	2611.26–3389.34	0.221 *	0.130	0.094	<0.001	−0.078
14.32_622.6100	1033.86 ± 2059.86	615.18	625.14–1442.59	0.116	0.214 *	0.078	-0.050	-0.005
14.32_661.5383	1073.18 ± 403.47	1059.45	993.12–1153.23	−0.012	−0.246 *	0.076	−0.008	−0.117
14.78_689.5731	1320.5 ± 399.15	1305.61	1241.30–1399.69	−0.024	−0.198 *	0.078	−0.029	−0.122
14.78_726.6541	1780.26 ± 580.83	1676.95	1665.01–1895.51	−0.062	−0.257 **	0.085	−0.122	−0.169 †
15.28_769.6350	1077.5 ± 301.88	1055.25	1017.60–1137.40	−0.047	−0.207 *	0.090	−0.031	−0.100
15.47_868.7413	2594.49 ± 3121.00	1585.10	1975.22–3213.76	0.073	0.084	-0.066	0.194 †	0.103
15.56_740.6769	113,168.6 ± 64,628.7	97,674.80	100,344.90–125,992.30	−0.072	−0.073	0.036	−0.109	−0.170 †
15.71_754.6932	7955.3 ± 8201.05	5557.18	6328.04–9582.57	−0.095	−0.031	0.115	−0.092	−0.181 †
15.87_771.7168	1031.7 ± 764.12	810.34	880.09–1183.32	−0.101	−0.033	−0.026	−0.122	−0.203 *
16.52_902.8188	73,288.14 ± 38,359.7	63,212.06	65,676.74–80899.53	0.030	0.078	0.026	0.071	0.206 *
16.99_907.8456	13,893.17 ± 12,526.2	9875.59	11,407.69–16,378.64	0.020	0.179 †	−0.026	0.059	0.060
Fatty acids (rt_*m*/*z*)								
0.93_171.1083	506.85 ± 727.61	352.22	362.48–651.22	0.056	−0.011	−0.090	0.194 †	0.026
0.93_273.1837	520.63 ± 459.27	468.74	429.50–611.76	−0.019	−0.007	−0.107	0.254 *	0.167 †
0.93_323.1899	1142.52 ± 2251.64	782.33	695.74–1589.29	−0.015	0.037	−0.136	0.227 *	0.097
0.93_407.2098	819.08 ± 684.47	583.53	683.26–954.89	−0.056	−0.053	−0.079	0.207 *	0.087
0.98_325.2054	3374.41 ± 5100.99	2519.93	2362.26–4386.56	0.012	0.022	−0.082	0.244 *	0.151
0.98_327.2215	6347.29 ± 5617.66	5370.78	5232.62–7461.95	0.023	0.034	−0.137	0.259 **	0.174 †
0.98_329.2375	20,982.0 ± 22,331.7	16,817.50	16,550.94–25,413.12	−0.014	−0.045	−0.167 †	0.209 *	0.171 †
1.02_329.2911	362.12 ± 591.52	262.90	244.75–479.49	−0.063	−0.053	−0.105	0.145	0.198 *
1.02_349.2050	826.74 ± 584.95	612.46	710.68–942.81	−0.027	0.007	−0.039	0.209 *	0.202 *
1.02_351.2207	1652.86 ± 1029.32	1338.27	1448.62–1857.10	0.002	0.003	−0.035	0.166 †	0.170 †
1.02_353.2364	1791.5 ± 1055.21	1423.75	1582.12–2000.87	0.020	0.026	−0.027	0.138	0.197 *
1.05_309.2112	5346.07 ± 3360.59	4604.94	4679.25–6012.88	0.096	0.139	−0.085	0.186 †	0.262 **
1.05_311.2272	17,455.4 ± 14,683.2	13,376.1	14,542.00–20,368.90	0.120	0.142	−0.082	0.117	0.282 **
1.05_311.2791	324.31 ± 404.65	208.03	244.02–404.60	0.108	0.151	−0.041	0.065	0.293 **
1.05_621.4340	905.06 ± 839.31	620.07	738.52–1071.59	0.135	0.201 *	−0.059	0.179	0.297 **
1.09_313.2398	1556.57 ± 1346.65	1192.46	1289.37–1823.78	0.050	0.174 †	−0.147	0.216*	0.289 **
1.09_335.2255	1289.6 ± 3151.7	717.62	664.24–1914.97	0.068	0.110	0.013	0.105	0.219 *
1.21_293.2166	8526.69 ± 6753.36	6641.14	7186.68–9866.70	0.031	0.141	−0.081	0.082	0.204 *
1.21_295.2321	9446.96 ± 14,642.01	5565.58	6541.67–12,352.25	0.080	0.164	−0.091	0.119	0.212 *
1.21_343.2241	4241.96 ± 8698.74	2599.43	2515.94–5967.98	−0.054	−0.001	−0.165	0.102	0.189 †
1.48_199.1758	3088.96 ± 1347.67	2693.66	2821.56–3356.37	−0.012	−0.085	0.039	−0.243 *	−0.233 *
1.52_249.1907	290.51 ± 208.75	257.44	249.09–331.93	0.034	0.057	0.201 *	−0.101	0.023
1.98_227.2067	1298.41 ± 862.33	990.62	1127.30–1469.51	0.007	−0.053	−0.052	−0.187 †	−0.250 *
2.72_255.2380	10,336.7 ± 12,253.1	6845.15	7905.46–12,768.01	0.031	−0.041	0.049	−0.251*	−0.207 *
2.79_537.4898	8384.1 ± 7892.01	5767.38	6818.16–9950.05	0.011	−0.028	0.056	−0.214 *	−0.192 †
3.84_283.2688	4498.68 ± 4366.43	3357.58	3632.29–5365.07	0.033	0.04	0.124	−0.168 †	−0.142
9.20_559.4733	1283.72 ± 1018.93	1028.39	1318.82–2128.91	−0.027	0.026	−0.167 †	0.058	−0.051
1.71_301.2218 ^a^	7742.7 ± 7409.11	5024.96	6272.57–9212.82	−0.061	−0.059	0.159	-0.02	−0.013
1.91_327.2374 ^b^	58,488.0 ± 42,277.3	45,997.00	50,099.30–66,876.80	−0.122	−0.078	0.137	−0.06	−0.028

Abbreviation: SD, standard deviation. † *p* < 0.1, * p < 0.05, ** p < 0.01; rt_*m*/*z*: Retention time and mass-to-charge-ratio. ^a^ Eicosapentaenoic acid. ^b^ Docosahexaenoic acid. ^c^ Lower bound. ^d^ Upper bound.

**Table 5:** Multivariate analyses between PBDEs, lipids, and fatty acids combined and the five domains of the Bayley-III developmental score (n = 100) adjusted for mother’s age, mother’s education, family income, mother’s smoking during pregnancy, prepregnant BMI, parity number of birth, living period in Kaoping area, newborn’s sex, gestational age (weeks), birth weight, birth length, and head circumference.

Bayley-III Score Domain	Category	Compound Description (rt_*m*/*z*)	β Estimate	95% CI	*p*-Value
Cognitive QIC = 51.63	PBDE	BDE-209	−0.0068	−0.0125–−0.0011	0.0018
	Lipid	15.47_868.7413	−0.0018	−0.0026–−0.0009	0.0001
	Fatty acid	3.84_283.2688	0.0029	0.0010–0.0048	0.0030
Language QIC = 51.62	Lipid	13.54_617.5134	−0.0136	−0.0215–−0.0057	0.0008
	Lipid	15.47_868.7413	−0.0014	−0.0023–−0.0006	0.0004
Motor QIC = 50.08	Lipid	5.08_590.4762	0.0022	0.0006–0.0037	0.0050
	Lipid	14.32_661.5383	0.0135	0.0046–0.0222	0.0027
Social−emotional QIC = 48.41	Lipid	5.08_590.4762	0.0058	0.0030–0.0086	<0.0001
	Lipid	16.99_907.8456	−0.0008	−0.0013–−0.0002	0.0061
	Fatty acid	0.93_171.1083	−0.0415	−0.0691–−0.0139	0.0032
	Fatty acid	1.02_353.2364	0.0122	0.0035–0.0208	0.0055
	Fatty acid	1.09_335.2255	−0.0038	−0.0056–−0.0020	<0.0001
Adaptive behavior QIC = 50.68	Lipid	1.02_348.2749	−0.0124	−0.0172–−0.0075	<0.0001
	Lipid	1.05_173.1178	0.0885	0.0409–0.1362	0.0003
	Lipid	14.32_661.5383	−0.0256	−0.0430–−0.0082	0.0039

Abbreviation: QIC, quasilikelihood under the independence mode criterion (the goodness of fit statistic for generalized estimating equation models).

**Table 6:** Information for significant lipids.

Compound Description (rt_m/z) [Ion]	Category [Subclass]	Formula	Common Name	Lipid Map Link
1.02_348.2749 [M + H]^T5AB^	Prenol lipids [PR0104] ^a^	C_19_H_24_O_6_	gibberellin A1	https://www.lipidmaps.org/data/LMSDRecord.php?&LMID=LMPR0104170001
1.05_173.1178 [M + H]^T5AB^	Prenol lipids [PR0102] ^b^	C_10_H_20_O_2_	1α,3α,4β-p-menthane-3,8-diol	https://www.lipidmaps.org/data/LMSDRecord.php?&LMID=LMPR0102090049
5.08_590.4762 [M + H]^T5M, T5SE^	Glycerophospholipids [GP0102] ^c^	C_26_H_54_NO_7_P	PE(21:0/0:0)	https://www.lipidmaps.org/data/LMSDRecord.php?&LMID=LMGP01020046
13.54_617.5134 [M + H]^T5L^	Glycerolipids [GL0201] ^d^	C_39_H_68_O_5_	DG(18:2(9Z,12Z)/18:2(9Z,12Z)/0:0)	https://www.lipidmaps.org/data/LMSDRecord.php?&LMID=LMGL02010063
14.32_661.5383 [M + H]^T5M, T5AB^	Sphingolipids [SP0302] ^e^	C_36_H_73_N_2_O_6_P	PE-Cer(d14:1(4E)/20:0)	https://www.lipidmaps.org/data/LMSDRecord.php?&LMID=LMSP03020005
15.47_868.7413 [M + H]^T5L^	Glycerophospholipids [GP0101] ^f^	C_50_H_94_NO_8_P	PC(20:1(11Z)/22:2(13Z,16Z))	https://www.lipidmaps.org/data/LMSDRecord.php?&LMID=LMGP01011832
16.99_907.8456 [M + H]^T5SE^	Glycerolipids [GL0301] ^g^	C_59_H_102_O_6_	TG(18:3(9Z,12Z,15Z)/18:3(9Z,12Z,15Z)/20:0)[iso3]	https://www.lipidmaps.org/data/LMSDRecord.php?&LMID=LMGL03010653
0.93_171.1083 [M − H]^T5SE^	Fatty acyls [FA0101] ^h^	C_10_H_20_O_2_	capric acid	https://www.lipidmaps.org/data/LMSDRecord.php?&LMID=LMFA01010010
1.02_353.2364 [M − H]^T5SE^	Fatty acyls [FA0301] ^i^	C_20_H_34_O_5_	PGF2alpha	https://www.lipidmaps.org/data/LMSDRecord.php?&LMID=LMFA03010002
1.09_335.2255 [M − H]^T5SE^	Fatty acyls [FA0200] ^j^	C_18_H_28_O_3_	α-licanic acid	https://www.lipidmaps.org/data/LMSDRecord.php?&LMID=LMFA02000273
3.84_283.2688 [M − H]^T5C^	Fatty acyls [FA0101] ^h^	C_18_H_36_O_2_	octadecanoic acid	https://www.lipidmaps.org/data/LMSDRecord.php?&LMID=LMFA01010018

The superscription after [ion] indicates in which table or figure the significance is shown. ^T4C^: Table 4 Cognitive; ^T4L^: Table 4 Language; ^T4M^: Table 4 Motor; ^T4SE^: Table 4 Social–emotional; ^T4AB^: Table 4 Adaptive behavior; ^a^ [PR0104]: C20 isoprenoids (diterpenes); ^b^ [PR0102]: C10 isoprenoids (monoterpenes); ^c^ [GP0102]: 1-alkyl,2-acylglycerophosphocholines; ^d^ [GL0201]: Diacylglycerols; N-acylsphingosines (ceramides); ^e^ [SP0302]: Ceramide phosphoethanolamines; ^f^ [GP0101]: Diacylglycerophosphocholines; ^g^ [GL0301]: Triacylglycerols; ^p^ [GL0201]: Diacylglycerols; ^h^ [FA0101]: Straight-chain fatty acids; ^I^ [FA0301]: Prostaglandins; ^j^ [FA0200]: Other octadecanoids.

## Data Availability

All relevant data are within the manuscript.

## Supplementary Materials

The following are available online at https://www.mdpi.com/article/10.3390/ijerph182111501/s1, Table S1: Detailed information related to 101 significant lipids identified in the Spearman correlation analyses and RDA maps; Figure S1: Flowchart showing the recruitment, enrollment, and inclusion of study participants; Figure S2. Box-and-whisker plots of 30 PBDE compounds and ΣPBDEs; References [48,49,50,51,52,53,54,55,56] are cited in the supplementary materials.
