# Supplementary material for: Analysis of Polybrominated Diphenyl Ethers and Lipid Composition in Human Breast Milk and Their Correlation with Infant Neurodevelopment
Source: Int J Environ Res Public Health. 2021 Nov 1;18(21):11501. doi: 10.3390/ijerph182111501 (PMC8583092; doi:10.3390/ijerph182111501)
Supplement: Supplementary file 1 [file ijerph-18-11501-s001.zip › ijerph-1399739-supplementary.pdf]

Analysis of Polybrominated Diphenyl Ethers and Lipid Composition in Human Breast Milk and Their Correlation with Infant Neurodevelopment

Ming-Hsien Tsai[1,2,3], How-Ran Chao[3,4,5,*,†], Wen-Li Hsu[2,3,6], Ching-Chung Tsai[7,8], Chu-Wen Lin[1], and Chu-Huang Chen[11,12,13,*]

[1]Department of Child Care, College of Humanities and Social Sciences, National Pingtung University of Science and Technology, No.1, Shuefu Road, Pingtung 91201, Taiwan; alantsai@mail.npust.edu.tw, cwlin@mail.npust.edu.tw
[2]Regenerative Medicine and Cell Therapy Research Center, Kaohsiung Medical University, No.100, Shin-Chuan 1st Road, Sanmin Dist., Kaohsiung 80708, Taiwan; alantsai@mail.npust.edu.tw, 1098129@kmuh.org.tw
[3]Emerging Compounds Research Center, General Research Service Center, National Pingtung University of Science and Technology, No.1, Shuefu Road, Pingtung 91201, Taiwan; hrchao@mail.npust.edu.tw, 1098129@kmuh.org.tw, 02791@ptch.org.tw
[4]Department of Environmental Science and Engineering, College of Engineering, National Pingtung University of Science and Technology, No.1, Shuefu Road, Pingtung 91201, Taiwan; hrchao@mail.npust.edu.tw, arcegarean@gmail.com
[5]Institute of Food Safety Management, College of Agriculture, National Pingtung University of Science and Technology, No.1, Shuefu Road, Pingtung 91201, Taiwan; hrchao@mail.npust.edu.tw
[6]Department of Dermatology, Kaohsiung Municipal Ta-Tung Hospital, Kaohsiung Medical University Hospital, Kaohsiung Medical University, No. 68, Jhonghua 3rd Rd, Kaohsiung 80145, Taiwan; 1098129@kmuh.org.tw
[7]Department of Pediatrics, E-Da Hospital, No.1, Yi-Da Road, Kaohsiung 82445, Taiwan; u101130@gmail.com
[8]School of Medicine, College of Medicine, I-Shou University, No. 8, Yi-Da Road, Kaohsiung 82445, Taiwan; u101130@gmail.com
[9]Vascular and Medicinal Research, Texas Heart Institute, 6770 Bertner Avenue, Houston, Texas 77030, USA; cchen@texasheart.org
[10]New York Heart Research Foundation, 200 Old Country Road, Mineola, New York, 11501, USA; cchen@texasheart.org
[11]Institute for Biomedical Sciences, Shinshu University, 3-1-1 Asahi Matsumoto Nagano 390-8621 Japan; cchen@texasheart.org

* Equal contributions.
† Correspondence: hrchao@mail.npust.edu.tw; Tel.: +886-8-7703202 #7514

Table of Contents

Appendix
Methods
Table S1. Detailed information related to 101 significant lipids identified in the Spearman correlation analyses and RDA maps.
Figure S1. Flowchart showing the recruitment, enrollment, and inclusion of study participants.
Figure S2. Box-and-whisker plots of 30 PBDE compounds and ΣPBDEs.
References

Appendix

Polybrominated diphenyl ethers (PBDEs) in the environment and their health effects on humans

PBDEs have long been used as additive brominated flame-retardants in a variety of products such as electronic equipment, textiles, foams, and plastics [1]. The PBDE formulations seeded into these products are sold commercially as technical mixtures of penta-BDE, octa-BDE, and deca-BDE [2]. However, the use and production of these mixtures have been banned and limited in some countries because of their persistence and resistance to degradation [3]. Owing to these chemical characteristics, PBDEs have been labeled as persistent organic pollutants (POPs) and were listed as the first brominated POPs under Annex A of the Stockholm Convention (StockholmConvention 2009). As POPs, PBDEs continue to persist ubiquitously in the environment, even after limitations of their use were implemented. At the present time, PBDEs can be detected globally in various environmental matrices such as soil, water, and air [4–6]. Furthermore, PBDEs have been found to accumulate in regions where they have never been used, such as in the Antarctic, due to their long-range atmospheric transport potential [7,8]. Moreover, detectable levels of PBDEs have been reported in indoor microenvironments (eg, homes, offices, cars) where PBDE-containing commercial products are most commonly located. PBDEs are not chemically bound into household products (eg, sofas, electronic equipment, furniture), which allows them to be easily dispersed into indoor air and dust [9,10]. In addition to the presence of PBDEs in the environment, PBDEs are also detected in tissues of animals and humans. Being lipophilic compounds, PBDEs accumulate in the lipid-rich tissues of humans including breast milk, cord blood, placenta, and adipose tissue [11–14]. Among these tissues, breast milk has received a great deal of attention because of the fact that breastfed infants of lactating mothers with a high body burden of PBDE may be at greater risk of adverse health effects. Mothers can be exposed to PBDEs through diverse pathways such as dust ingestion, dietary intake, inhalation, and absorption from dermal contact [15]. PBDEs are then passed on from mother to offspring during pregnancy and through breastfeeding [16] during the infant's early developmental stages.

The common presence of PBDEs has raised public concern because of the negative impact that they have not only on the environment, but also on humans. Evidence from animal and human studies has indicated that PBDEs may induce thyroid hormone disruption, impair neurodevelopment, cause behavioral changes, affect reproductive and immune systems, and, for some congeners, possibly cause cancer in animals (ATSDR 2015). Currently, there is a substantial amount of evidence linking neurodevelopment with PBDE exposure in humans, with a focus on infants and children due to higher PBDE concentrations observed in these age groups than in adults [17]. Chao et al. [18] and Gascon et al. [19] reported that postnatal exposure to PBDEs in breast milk, particularly the congener BDE-209, could potentially delay neurologic and mental development in nursing infants at 8 to 18 months of age. Prenatal exposure to PBDEs may also affect cognition and delay the adaptive behavior development of infants [20]. For example, the congener BDE-47 in serum was found to be associated with poorer cognitive abilities, as indicated by the lower intelligence quotient (IQ) observed in 5- and 8-year-old children who were exposed prenatally [21]. In addition, an association between PBDEs and neurodevelopment was reported in a cohort study of children 1 to 6 years of age, whereby higher concentrations of BDE-47, BDE-99, and BDE-100 in cord blood were associated with lower scores on tests of mental development [22]. Results from such epidemiologic studies have raised the possibility that PBDEs may be a contributing factor to the development of far more serious neurodevelopmental disorders such as attention deficit hyperactivity disorder and autism spectrum disorder. Gascon et al. [19] reported that postnatal exposure to BDE-47 was significantly associated with the increased risk of symptoms of attention deficit disorder but not of hyperactivity. Additionally, Adgent et al. [23] reported that BDE-28 and BDE-99 in breast milk were associated with more anxious behavior and increased withdrawal in toddlers at 36 months of age. In contrast, no associations were found between PBDEs and autism [24].

Materials and Methods

Study participants

The participants in this study were pairs of healthy mothers and infants recruited from local hospitals in southern Taiwan between April 2007 and March 2011. We recruited mother-infant pairs with background-level organohalogen exposure to study its correlation with adverse health effects including neurologic toxicity. The study protocol was reviewed and approved by the institutional review boards of the Human Ethical Committees of Pingtung Christian Hospital (PCH) in 2007 (NO: IRB021). The participants were randomly recruited pregnant women undergoing routine health checks in the obstetric clinics of local hospitals, as described previously [25,26]. The participants were initially selected according to the following criteria: an agreement to donate breast milk; living in southern Taiwan for a minimum of three years; no smoking during pregnancy; a plan to breastfeed for at least two months; and a willingness to strictly follow our protocol. The process of enrolling the participants is described briefly as follows. More than 500 pregnant women were recruited, and 358 participants were enrolled. Four pregnant participants were excluded because of medical concerns following the obstetrician's recommendations. Of the 354 healthy pregnant participants, 265 completely answered the detailed questionnaire. A group of 145 participants voluntarily donated breast milk. Of these women, seven did not donate sufficient breast milk samples for further chemical analysis and were excluded. Therefore, a total of 138 mothers provided sufficient breast milk (>90 mL) for the chemical analysis of PBDEs. Breast milk was collected at childbirth and within a month after delivery. The parameters of birth outcomes including gestational age, birth weight, birth length, and head circumference were recorded at birth by the pediatricians.

After delivery, if mothers donated sufficient breast milk and PBDEs were detected in the breast milk, their nursing infants were enrolled in our cohort for a follow-up evaluation at the ages of 8 to 12 months. Postcards were sent to this cohort (n=138) inviting them to join the follow-up program. The mothers who were contacted by telephone had agreed to participate in the program and were asked to bring their infants to the Department of Pediatrics in PCH to assess infant development. More than 95% of the cohort participated in this follow-up program and were reviewed and evaluated by pediatricians. Twenty-eight infants were excluded because of exclusive formula feeding, and four participants did not complete the neurodevelopment assessment. A total of 100 mother-infant pairs was included in the present study on the basis of exclusive or partial breastfeeding during the first six months of lactation. Lipids and lipid metabolites in the breast milk of mothers from the 100 mother-infant pairs were analyzed by performing additional chemical analysis.

Breast milk sample collection

Breast milk samples were collected in chemical-free glass bottles and frozen in the subjects' home refrigerators (-4°C) within one month after delivery. When the milk samples were more than 90 mL, the mothers notified us and our team members immediately transferred the samples to our laboratory at the Department of Environmental Science and Engineering, National Pingtung University of Science and Technology, to be stored at -20°C in a freezer. For the chemical analysis of PBDEs, 25 mL of the milk samples were transported to the Supermicro Mass Research and Technology Center at Cheng Shiu University in southern Taiwan.

Chemical analysis of PBDEs

Breast milk was analyzed for PBDEs according to the analytical method described previously [18,26]. The 30 PBDE congeners including BDE-7, -15, -17, -28, -47, -49, -66, -71, -77, -85, -99, -100, -119, -126, -138, -139, -140, -153, -154, -156, -183, -184, -191, -196, -197, -203 -206, -207, -208, and -209 were analyzed by using high-resolution gas chromatography with high-resolution mass spectrometry (HRGC/HRMS). The mixtures of PBDE standards were obtained from Cambridge Isotope Laboratories (Andover, MA, USA). PBDE standards labeled with the $^{13}C_{12}$ isotopes were purchased from Wellington Laboratories (Guelph, Canada). The highest-quality sodium sulfate, alumina oxide, potassium oxalate, and silica gel were obtained from Merck (Darmstadt, Germany), Tedia (Fairfield, OH, USA), and Sigma-Aldrich (St. Louis, MO, USA).

Breast milk samples (15 mL) were combined with eight internal standards, including $^{13}C_{12}$-labeled

BDE-28, -47, -99, -153, -183, -197, -207, and -209, and were extracted by means of sonication with a mixture of *n*-hexane (15 mL) and acetone (45 mL) for 20 minutes. The extract was then centrifuged at 2,000 rpm for 15 minutes at 20°C. This procedure was repeated at least three times. After sample extraction, the lipid content of the breast milk was determined by using the gravimetric method. The extract was concentrated and dissolved in *n*-hexane and was treated with concentrated sulfuric acid for the cleanup procedure by passage through a multi-column system. The eluate was concentrated to near-dryness, re-dissolved in *n*-hexane (100 μL), and then transferred to a vial (1 mL) under a gentle nitrogen stream. The final eluate was analyzed with HRGC/HRMS (Hewlett-Packard GC 6970/Micromass Autospec Ultima, GC and MS were from Hewlett-Packard, Palo Alto, CA, USA and Waters, Milford, MA, USA, respectively). Quantification was performed by using internal/external standard mixtures with the isotope dilution method. Eight $^{13}C_{12}$-labeled PBDE internal standards were added to the breast milk before extraction to ensure recovery in the chemical analysis process. The blank tests including solvent and glassware blanks were regularly checked to ensure quality control during the experiments. To further ensure quality control, a set of PBDE standards, a set of blanks, and a pool of 4 breast milk samples were confirmed in each batch of approximately 10 samples. Limits of detection (LODs) were predetermined so that the signal-to-noise ratios for both ions of a specific congener would be above three. The MDLs of breast milk PBDEs were measured as 0.980-17.4 pg/g lipid for BDE-7 to BDE-208 and 115 pg/g lipid for BDE-209 only. For measurements below the MDLs, PBDE concentrations were recognized as half of the MDLs.

Chemical analysis of lipids and lipid metabolites

The lipids and lipid metabolites were extracted from the breast milk of the selected participants (n=100) by using the Oasis® HLB (Waters Corporation, MA, USA) solid phase extract (SPE) method. Briefly, the SPE column resin was conditioned with 50% methanol (Sigma Aldrich) for 20 minutes. Human breast milk (1 mL) was added into the SPE column and incubated for 10 minutes. The sample was eluted with 500 μL isopropanol alcohol and then dried by N_2 gas for 30 minutes. Finally, the sample was dissolved in 200 μL of lipid solution, which consisted of isopropanol/acetonitrile/water (2:1:1). Lipids and lipid metabolites were quantified by using liquid chromatography data-independent, parallel-fragmentation mass spectrometry (LC/MS^E). Briefly, lipids and lipid metabolites were chromatographically separated on an ACQUITY ultra performance liquid chromatography separation system (Waters Corporation, MA, USA) incorporated with a CSH™, 130Å, 1.7 μm, 1.0 mm × 10 cm C-18 column under gradient conditions at a flow rate of 0.1 mL/min for 20 minutes at 55°C. The mobile phase A was composed of 10 mM NH_4HCO_2 in acetonitrile/H_2O (60:40) and 0.1% formic acid (0.1% v/v), and mobile phase B was composed of 10 mM NH_4HCO_2 in isopropanol/acetonitrile (90:10) and 0.1% formic acid (0.1% v/v) for molecule protonation. Mass spectrometry was performed on a Xevo G2 qTof (Waters Corporation, MA, USA) instrument equipped with an electrospray ionization probe (ESI, Waters) interface with 3KV for positive mode (ES+) and 2KV for negative mode (ES-). The mass spectrometer was operated in the data-independent collection mode (MS^E). Parallel ion fragmentation was programmed to switch between low (4 eV) and high (35–55 eV) energies in the collision cell, and data were collected from 200 to 1600 *m*/*z* by utilizing leucine (Sigma Aldrich, *m*/*z* 556.2771 for ES+ and 554.2704 for ES-) as the separate data channel lock mass calibrant. Mass spectrometry data were imported, processed, and identified by using the LIPID MAPS Structure Database (LMSD) 2019-07-11 updated with Nonlinear Progenesis QI software (Waters Corporation, MA, USA). The identified compounds' normalized abundance and raw abundance were exported from QI software and prepared for further correlation analysis and univariate and multivariate analyses.

Neurodevelopmental test

Our selected infant cohort was reviewed and evaluated by pediatricians, and infant neurodevelopment was assessed at the ages of 8 to 12 months by infant psychometrists. The Bayley Scales of Infants and Toddlers Development, Third Edition (Bayley-III), were used to examine infants'

neurologic and neurobehavioral development from birth to four years of age (the best period for Bayley-III assessment is from six months to 2.5 years). The Bayley-III has five domains including three major parts, which are the cognitive, language (receptive and expressive communication), and motor (fine and gross motor) scales, which were assessed by the infant psychometrist in combination with two parent-report questionnaires. The questionnaires were answered by parents who agreed to assess social-emotional and adaptive behavior scales [18,27]. The Bayley-III composite scores provided developmental quotients, including raw scores and chronological age, and generated continuous outcome scores for the cognitive, language, motor, social-emotional, and adaptive behavior scales. The Bayley-III composite scores are qualitatively interpreted to determine an infant or toddler's level of performance in terms of neurodevelopment and are categorized into the following different levels: very superior (130 and above), superior (120-129), high average (110-119), average (90-109), low average (80-89), borderline (70-79), or extremely low (69 and below). On the basis of these categories, scores that fall below 70 indicate poor performance. The Bayley-III scale is designed to help parents, pediatricians, and caregivers better understand a child's strengths and weaknesses in terms of mental and motor development.

Statistical analysis

Descriptive statistics, Spearman correlation, and univariate and multivariate analyses were conducted to investigate the associations between PBDEs, lipids, and fatty acids with the five domains Bayley-III scores. All statistical analyses were conducted by using Statistical Product and Service Solutions (SPSS) V13 and Statistical Analysis System (SAS) V9.4. Redundancy analyses (RDA) were used to investigate the canonical correlation between PBDEs, lipids, and fatty acids with the five domains Bayley-III scores by using the XLSTAT Microsoft Excel extension package (Addinsoft, New York, USA).

Information related to significant lipids and fatty acids

Table S1. Detailed information related to 101 significant lipids identified in the Spearman correlation analyses and RDA maps.

rt_m/z [Ion]	Category [Sub class]	Formula	Common Name	Link
0.74_854.3112 [M+H]	Prenol Lipids [PR0104][a]	$C_{47}H_{51}NO_{14}$	Paclitaxel	https://www.lipidmaps.org/data/LMSDRecord.php?&LMID=LMPR0104390001
0.83_309.2057 [M+H]	Prenol Lipids [PR0104][a]	$C_{20}H_{36}O_2$	Sclareol	https://www.lipidmaps.org/data/LMSDRecord.php?&LMID=LMPR0104030010
0.86_884.1628 [M+H]	Polyketides [PK1201][b]	$C_{40}H_{45}O_{21}$	Pelargonidin 3-rutinoside-7-(6-(p-hydroxybenzoyl)glucoside)	https://www.lipidmaps.org/data/LMSDRecord.php?LMID=LMPK12010082
0.93_353.2246 [M+H]	Prenol Lipids [PR0104][a]	$C_{20}H_{32}O_5$	Grayanotoxin II	https://www.lipidmaps.org/data/LMSDRecord.php?&LMID=LMPR0104180001
0.93_365.1058 [M+H]	Prenol Lipids [PR0104][a]	$C_{20}H_{30}O_4$	terpentecin	https://www.lipidmaps.org/data/LMSDRecord.php?&LMID=LMPR0104040002
1.02_335.2180 [M+H]	Sterol Lipids [ST0203][c]	$C_{21}H_{34}O_3$	21-hydroxyallopregnanolone	https://www.lipidmaps.org/data/LMSDRecord.php?&LMID=LMST02030132
1.02_348.2749 [M+H]	Prenol Lipids [PR0104][a]	$C_{19}H_{24}O_6$	Gibberellin A1	https://www.lipidmaps.org/data/LMSDRecord.php?&LMID=LMPR0104170001
1.02_367.1932 [M+H]	Prenol Lipids [PR0104][a]	$C_{20}H_{30}O_6$	Ingol	https://www.lipidmaps.org/data/LMSDRecord.php?&LMID=LMPR0104310002
1.02_369.2070 [M+H]	Prenol Lipids [PR0103][d]	$C_{20}H_{30}O_6$	Rhodojaponin III	https://www.lipidmaps.org/data/LMSDRecord.php?&LMID=LMGP10050001
1.05_173.1178 [M+H]	Prenol Lipids [PR0102][e]	$C_{10}H_{20}O_2$	1α,3α,4β-p-menthane-3,8-diol	https://www.lipidmaps.org/data/LMSDRecord.php?&LMID=LMPR0102090049

1.05_195.1378 [M+Na]	Prenol Lipids [PR0102][e]	$C_{10}H_{20}O_2$	1α,3α,4β-p-menthane-3,8-diol [M+Na]	https://www.lipidmaps.org/data/LMSDRecord.php?&LMID=LMPR0102090049
1.05_213.1488 [M+Na]	Prenol Lipids [PR0107][f]	$C_{13}H_{18}O$	trans-β-damascenone [M+Na]	https://www.lipidmaps.org/data/LMSDRecord.php?&LMID=LMPR01070304
1.05_311.2221 [M+H]	Prenol Lipids [PR0103][d]	$C_{17}H_{26}O_5$	Botrydial	https://www.lipidmaps.org/data/LMSDRecord.php?&LMID=LMPR0103640001
1.05_349.1826 [M+H]	Prenol Lipids [PR0104][a]	$C_{20}H_{28}O_5$	Novaxenicins A	https://www.lipidmaps.org/data/LMSDRecord.php?&LMID=LMPR0104420001
1.05_351.1958 [M+H]	Prenol Lipids [PR0103][d]	$C_{19}H_{26}O_6$	Alatolide	https://www.lipidmaps.org/data/LMSDRecord.php?&LMID=LMPR0103090013
1.09_293.2119 [M+H]	Sterol Lipids [ST0202][g]	$C_{19}H_{32}O_2$	3α-androstanediol	https://www.lipidmaps.org/data/LMSDRecord.php?&LMID=LMST02020052
1.09_330.2644 [M+H]	Sphingolipids [SP0108][h]	$C_{19}H_{39}NO_3$	Penaresidin A	https://www.lipidmaps.org/data/LMSDRecord.php?&LMID=LMSP01080045
1.21_277.2170 [M+H]	Sterol Lipids [ST0201][i]	$C_{18}H_{28}O_2$	19-norandrosterone	https://www.lipidmaps.org/data/LMSDRecord.php?&LMID=LMST02010042
1.98_417.2979 [M+H]	Sterol Lipids [ST0101][j]	$C_{27}H_{44}O_3$	2,22,25-trideoxyecdysone	https://www.lipidmaps.org/data/LMSDRecord.php?&LMID=LMST01010179
2.10_331.2849 [M+H]	Sterol Lipids [ST0302][k]	$C_{22}H_{34}O_2$	1α-hydroxy-23,24,25,26,27-pentanorvitamin D3	https://www.lipidmaps.org/data/LMSDRecord.php?&LMID=LMST03020009
2.10_331.2849 [M+H]	Sterol Lipids [ST0302][k]	$C_{32}H_{50}O_7$	16-Glutaryloxy-1alpha,25-dihydroxyvitamin D3	https://www.lipidmaps.org/data/LMSDRecord.php?&LMID=LMST03020634
3.00_547.3432 [M+H]	Sphingolipids [SP00][L]	$C_{33}H_{67}NO_5S$	Sulfobacin B	https://www.lipidmaps.org/data/LMSDRecord.php?&LMID=LMSP00000020

3.88_419.3163 [M+H]	Sterol Lipids [ST0101]	$C_{27}H_{46}O_3$	Dormatinol	https://www.lipidmaps.org/data/LMSDRecord.php?&LMID=LMST01010101
5.08_590.4762 [M+H]	Glycerophospholipids [GP0102][m]	$C_{26}H_{54}NO_7P$	PE(21:0/0:0)	https://www.lipidmaps.org/data/LMSDRecord.php?&LMID=LMGP01020046
5.24_524.3729 [M+H]	Glycerophospholipids [GP0409][n]	$C_{60}H_{111}O_{11}P$	SLBPA(54:3)	https://www.lipidmaps.org/data/LMSDRecord.php?&LMID=LMGP04090002
5.24_1061.7368 [M+H]	Sterol Lipids [ST0101][j]	$C_{29}H_{46}O_7$	2-deoxy-20-hydroxy-5α-ecdysone 3-acetate	https://www.lipidmaps.org/data/LMSDRecord.php?&LMID=LMST01010191
6.05_507.4033 [M+H]	Glycerolipids [GL0301][o]	$C_{66}H_{102}O_6$	TG(19:1_22:6_22:6)	https://www.lipidmaps.org/data/LMSDRecord.php?&LMID=LMGL03013150
6.05_991.8181 [M+H]	Glycerolipids [GL0201][p]	$C_{33}H_{58}O_5$	DG(12:0/18:3(9Z,12Z,15Z)/0:0)[iso2]	https://www.lipidmaps.org/data/LMSDRecord.php?&LMID=LMGL02010338
6.13_706.5438 [M+H]	Glycerophospholipids [GP0101]	$C_{38}H_{76}NO_8P$	PC(10:0/20:0)	https://www.lipidmaps.org/data/LMSDRecord.php?&LMID=LMGP01010395
7.80_535.4360 [M+H]	Glycerolipids [GL0301][q]	$C_{67}H_{124}O_6$	TG(20:0/22:0/22:3(10Z,13Z,16Z))[iso6]	https://www.lipidmaps.org/data/LMSDRecord.php?&LMID=LMGL03012110
7.80_1047.8799 [M+Na]	Glycerolipids [GL0201][p]	$C_{33}H_{60}O_5$	DG(15:1(9Z)/15:1(9Z)/0:0)	https://www.lipidmaps.org/data/LMSDRecord.php?&LMID=LMGL02010327
9.11_537.5353 [M+H]	Glycerophospholipids [GP0101][r]	$C_{28}H_{54}NO_8P$	PC(18:1(9E)/2:0)	https://www.lipidmaps.org/data/LMSDRecord.php?&LMID=LMGP01010878
10.16_564.4132 [M+H]	Glycerolipids [GL0201][p]	$C_{37}H_{64}O_5$	DG(17:2(9Z,12Z)/17:2(9Z,12Z)/0:0)	https://www.lipidmaps.org/data/LMSDRecord.php?&LMID=LMGL02010028
10.20_523.4734 [M+H]	Glycerolipids [GL0201]	$C_{32}H_{58}O_5$	DG(12:0/17:2(9Z,12Z)/0:0)[iso2]	https://www.lipidmaps.org/data/LMSDRecord.php?&LMID=LMGL02010334

11.09_615.4976 [M+H]	Glycerolipids [GL0201][p]	$C_{39}H_{66}O_5$	DG(18:2(9Z,12Z)/18:3(9Z,12Z,15Z)/0:0)[iso2]	https://www.lipidmaps.org/data/LMSDRecord.php?&LMID=LMGL02010071
11.90_565.5672 [M+H]	Glycerolipids [GL0201][p]	$C_{35}H_{64}O_5$	DG(16:1(9Z)/16:1(9Z)/0:0)	https://www.lipidmaps.org/data/LMSDRecord.php?&LMID=LMGL02010011
13.54_617.5134 [M+H]	Glycerolipids [GL0201][p]	$C_{39}H_{68}O_5$	DG(18:2(9Z,12Z)/18:2(9Z,12Z)/0:0)	https://www.lipidmaps.org/data/LMSDRecord.php?&LMID=LMGL02010063
13.85_801.6849 [M+H]	Sphingolipids [SP0301][s]	$C_{46}H_{93}N_2O_6P$	SM(d16:1/25:0)	https://www.lipidmaps.org/data/LMSDRecord.php?&LMID=LMSP03010076
14.11_815.7014 [M+H]	Sphingolipids [SP0301][s]	$C_{47}H_{95}N_2O_6P$	SM(d18:1/24:0)	https://www.lipidmaps.org/data/LMSDRecord.php?&LMID=LMSP03010008
14.16_579.5402 [M+H]	Glycerolipids [GL0201][p]	$C_{36}H_{66}O_5$	DG(16:1(9Z)/17:1(9Z)/0:0)[iso2]	https://www.lipidmaps.org/data/LMSDRecord.php?&LMID=LMGL02010016
14.32_622.6100 [M+H]	Sphingolipids [SP0201][t]	$C_{40}H_{79}NO_3$	Cer(d18:1/22:0)	https://www.lipidmaps.org/data/LMSDRecord.php?&LMID=LMSP02010008
14.32_661.5383 [M+H]	Sphingolipids [SP0302][u]	$C_{36}H_{73}N_2O_6P$	PE-Cer(d14:1(4E)/20:0)	https://www.lipidmaps.org/data/LMSDRecord.php?&LMID=LMSP03020005
14.78_684.6147 [M+H]	Sphingolipids [SP0203][v]	$C_{42}H_{85}NO_5$	Cer(t18:0/24:0(2OH))	https://www.lipidmaps.org/data/LMSDRecord.php?&LMID=LMSP02030002
14.78_689.5731 [M+H]	Sphingolipids [SP0301][s]	$C_{38}H_{77}N_2O_6P$	SM(d16:1/17:0)	https://www.lipidmaps.org/data/LMSDRecord.php?&LMID=LMSP03010037
14.78_726.6541 [M+H]	Sphingolipids [SP0203][v]	$C_{44}H_{87}NO_6$	Cer(t18:1(8E)/26:0(2OH[R])(3OH))	https://www.lipidmaps.org/data/LMSDRecord.php?&LMID=LMSP02030027
15.28_769.6350 [M+H]	Glycerolipids [GL0301][o]	$C_{44}H_{81}O_8P$	TG(12:0/12:0/22:5(7Z,10Z,13Z,16Z,19Z))[iso3]	https://www.lipidmaps.org/data/LMSDRecord.php?&LMID=LMGL03012657

15.47_868.7413 [M+H]	Glycerophospholipids [GP0101][r]	$C_{50}H_{94}NO_8P$	PC(20:1(11Z)/22:2(13Z,16Z))	https://www.lipidmaps.org/data/LMSDRecord.php?&LMID=LMGP01011832
15.47_894.7612 [M+H]	Glycerophospholipids [GP0101][r]	$C_{52}H_{96}NO_8P$	PC(22:0/22:4(7Z,10Z,13Z,16Z))	https://www.lipidmaps.org/data/LMSDRecord.php?&LMID=LMGP01012002
15.56_740.6769 [M+H]	Sphingolipids [SP0501][w]	$C_{43}H_{81}NO_8$	GlcCer(d15:2(4E,6E)/22:0)	https://www.lipidmaps.org/data/LMSDRecord.php?&LMID=LMSP0501AA60
15.71_754.6932 [M+H]	Sphingolipids [SP0501][w]	$C_{44}H_{83}NO_8$	GlcCer(d18:2/20:0)	https://www.lipidmaps.org/data/LMSDRecord.php?&LMID=LMSP0501AA34
15.81_858.7555 [M+H]	Glycerophospholipids [GP0102]	$C_{50}H_{100}NO_7P$	PC(O-22:0/20:1(11Z))	https://www.lipidmaps.org/data/LMSDRecord.php?&LMID=LMGP01020250
15.87_771.7168 [M+H]	Sphingolipids [SP0301][s]	$C_{44}H_{87}N_2O_6P$	SM(d18:2/21:0)	https://www.lipidmaps.org/data/LMSDRecord.php?&LMID=LMSP03010064
16.37_888.7954 [M+H]	Glycerophospholipids [GP0101][r]	$C_{51}H_{102}NO_8P$	PC(25:0/18:0)	https://www.lipidmaps.org/data/LMSDRecord.php?&LMID=LMGP01011175
16.52_902.8188 [M+H]	Glycerophospholipids [GP0101][r]	$C_{52}H_{104}NO_8P$	PC(18:0/26:0)	https://www.lipidmaps.org/data/LMSDRecord.php?&LMID=LMGP01010828
16.99_907.8456 [M+H]	Glycerolipids [GL0301][o]	$C_{59}H_{102}O_6$	TG(18:3(9Z,12Z,15Z)/18:3(9Z,12Z,15Z)/20:0)[iso3]	https://www.lipidmaps.org/data/LMSDRecord.php?&LMID=LMGL03010653
0.78_365.2073 [M-H]	Fatty Acyls [FA0301][x]	$C_{21}H_{34}O_5$	15-methyl-15S-PGD2	https://www.lipidmaps.org/data/LMSDRecord.php?&LMID=LMFA03010050
0.90_243.1286 [M-H]	Fatty Acyls [FA0117][y]	$C_{13}H_{24}O_4$	2-methyl-dodecanedioic acid	https://www.lipidmaps.org/data/LMSDRecord.php?&LMID=LMFA01170010
0.93_171.1083 [M-H]	Fatty Acyls [FA0101][z]	$C_{10}H_{20}O_2$	Capric acid	https://www.lipidmaps.org/data/LMSDRecord.php?&LMID=LMFA01010010

0.93_273.1837 [M-H]	Fatty Acyls [FA0103][α]	$C_{18}H_{26}O_2$	3,6,9,12,15-octadecapentaenoic acid	https://www.lipidmaps.org/data/LMSDRecord.php?&LMID=LMFA01030359
0.93_323.1899 [M-H]	Fatty Acyls [FA0103][α]	$C_{22}H_{28}O_2$	4,7,10,13-Docosatetraynoic acid	https://www.lipidmaps.org/data/LMSDRecord.php?&LMID=LMFA01030680
0.93_367.2138 [M-H]	Fatty Acyls [FA0301][x]	$C_{20}H_{32}O_6$	PGG2	https://www.lipidmaps.org/data/LMSDRecord.php?&LMID=LMFA03010009
0.93_407.2098 [M-H]	Fatty Acyls [FA0301][x]	$C_{23}H_{36}O_6$	15R-PGE2 methyl ester, 15-acetate	https://www.lipidmaps.org/data/LMSDRecord.php?&LMID=LMFA03010201
0.98_307.1955 [M-H]	Fatty Acyls [FA0203][β]	$C_{18}H_{28}O_4$	ent-9-L1-PhytoP	https://www.lipidmaps.org/data/LMSDRecord.php?&LMID=LMFA02030003
0.98_325.2054 [M-H]	Fatty Acyls [FA0200][γ]	$C_{18}H_{30}O_5$	2R-HpOTrE	https://www.lipidmaps.org/data/LMSDRecord.php?&LMID=LMFA02000031
0.98_327.2215 [M-H]	Fatty Acyls [FA0105][δ]	$C_{18}H_{32}O_5$	Malyngic acid	https://www.lipidmaps.org/data/LMSDRecord.php?&LMID=LMFA01050531
0.98_329.2375 [M-H]	Fatty Acyls [FA0200][γ]	$C_{18}H_{34}O_5$	9,12,13-TriHOME	https://www.lipidmaps.org/data/LMSDRecord.php?&LMID=LMFA02000014
0.98_345.2330 [M-H]	Fatty Acyls [FA0117][y]	$C_{18}H_{34}O_6$	Floionic acid	https://www.lipidmaps.org/data/LMSDRecord.php?&LMID=LMFA01170030
0.98_391.2148 [M-H]	Fatty Acyls [FA0103][α]	$C_{26}H_{48}O_2$	17,20-hexacosadienoic acid	https://www.lipidmaps.org/data/LMSDRecord.php?&LMID=LMFA01030133
0.98_393.2301 [M-H]	Fatty Acyls [FA0103][α]	$C_{26}H_{50}O_2$	9-hexacosenoic acid	https://www.lipidmaps.org/data/LMSDRecord.php?&LMID=LMFA01030094
1.02_329.2911 [M-H][T6S]	Fatty Acyls [FA0400][ε]	$C_{22}H_{34}O_2$	DPA	https://www.lipidmaps.org/data/LMSDRecord.php?&LMID=LMFA04000044

1.02_349.2050 [M-H]	Fatty Acyls [FA0103][α]	$C_{20}H_{30}O_5$	5S-Hp-18R-HEPE	https://www.lipidmaps.org/data/LMSDRecord.php?&LMID=LMFA03070034
1.02_351.2207 [M-H]	Fatty Acyls [FA0301][x]	$C_{20}H_{32}O_5$	PGE2	https://www.lipidmaps.org/data/LMSDRecord.php?&LMID=LMFA03010003
1.02_353.2364 [M-H]	Fatty Acyls [FA0301]	$C_{20}H_{34}O_5$	PGF2alpha	https://www.lipidmaps.org/data/LMSDRecord.php?&LMID=LMFA03010002
1.02_357.2086 [M-H]	Fatty Acyls [FA0103][α]	$C_{24}H_{38}O_2$	6Z,9Z,12Z,15Z,18Z-tetracosapentaenoic acid	https://www.lipidmaps.org/data/LMSDRecord.php?&LMID=LMFA01030820
1.02_369.2308 [M-H]	Fatty Acyls [FA0301][x]	$C_{20}H_{34}O_6$	6-keto-PGF1α	https://www.lipidmaps.org/data/LMSDRecord.php?&LMID=LMFA03010001
1.02_375.2202 [M-H]	Fatty Acyls [FA0403][ζ]	$C_{22}H_{32}O_5$	Resolvin D2	https://www.lipidmaps.org/data/LMSDRecord.php?&LMID=LMFA04030001
1.02_377.2357 [M-H]	Fatty Acyls [FA0301][x]	$C_{23}H_{38}O_4$	9-deoxy-9-methylene-16,16-dimethyl-PGE2	https://www.lipidmaps.org/data/LMSDRecord.php?&LMID=LMFA03010059
1.02_471.0770 [M-H]	Fatty Acyls [FA0312]	$C_{21}H_{29}IO_4$	iodovulone I	https://www.lipidmaps.org/data/LMSDRecord.php?&LMID=LMFA03120014
1.05_213.1546 [M-H]	Fatty Acyls [FA0105][δ]	$C_{12}H_{22}O_3$	12-hydroxy-10-dodecenoic acid	https://www.lipidmaps.org/data/LMSDRecord.php?&LMID=LMFA01050168
1.05_309.2112 [M-H]	Fatty Acyls [FA0200][γ]	$C_{18}H_{30}O_4$	9-HpOTrE	https://www.lipidmaps.org/data/LMSDRecord.php?&LMID=LMFA02000018
1.05_311.2272 [M-H]	Fatty Acyls [FA0200][γ]	$C_{18}H_{32}O_4$	8R,11S-DiHODE	https://www.lipidmaps.org/data/LMSDRecord.php?&LMID=LMFA02000064
1.05_311.2791 [M-H]	Fatty Acyls [FA0101][z]	$C_{20}H_{40}O_2$	Arachidic acid	https://www.lipidmaps.org/data/LMSDRecord.php?&LMID=LMFA01010020

1.05_333.2105 [M-H]	Fatty Acyls [FA0301][x]	$C_{20}H_{30}O_4$	PGB2	https://www.lipidmaps.org/data/LMSDRecord.php?&LMID=LMFA03010018
1.05_359.2229 [M-H]	Fatty Acyls [FA0400][ε]	$C_{22}H_{32}O_4$	17S-HpDHA	https://www.lipidmaps.org/data/LMSDRecord.php?&LMID=LMFA04000051
1.05_621.4340 [M-H]	Fatty Acyls [unavailable]	$C_{37}H_{66}O_7$	Bullatacinone	https://www.lipidmaps.org/data/LMSDRecord.php?&LMID=LMFA05000646
1.09_239.1697 [M-H]	Fatty Acyls [FA0106]	$C_{14}H_{24}O_3$	7-oxo-11E-Tetradecenoic acid	https://www.lipidmaps.org/data/LMSDRecord.php?&LMID=LMFA01060186
1.09_313.2398 [M-H]	Fatty Acyls [FA0200][γ]	$C_{18}H_{34}O_4$	10S-HpOME	https://www.lipidmaps.org/data/LMSDRecord.php?&LMID=LMFA02000059
1.09_335.2255 [M-H]	Fatty Acyls [FA0200][γ]	$C_{18}H_{28}O_3$	α-Licanic acid	https://www.lipidmaps.org/data/LMSDRecord.php?&LMID=LMFA02000273
1.14_291.2007 [M-H]	Fatty Acyls [FA0107][η]	$C_{16}H_{28}O_3$	10,11-Epoxy-7Z-hexadecenoic acid	https://www.lipidmaps.org/data/LMSDRecord.php?&LMID=LMFA01070026
1.17_267.2008 [M-H]	Fatty Acyls [FA0105][θ]	$C_{18}H_{30}O_3$	Agonandric acid	https://www.lipidmaps.org/data/LMSDRecord.php?&LMID=LMFA01050307
1.21_293.2166 [M-H]	Fatty Acyls [FA0200][γ]	$C_{18}H_{32}O_3$	9R-HODE	https://www.lipidmaps.org/data/LMSDRecord.php?&LMID=LMFA02000036
1.21_295.2321 [M-H]	Fatty Acyls [FA0400][ε]	$C_{22}H_{32}O_3$	14S-HDHA	https://www.lipidmaps.org/data/LMSDRecord.php?&LMID=LMFA04000058
1.21_343.2241 [M-H]	Fatty Acyls [FA0101][z]	$C_{12}H_{24}O_2$	Lauric acid	https://www.lipidmaps.org/data/LMSDRecord.php?&LMID=LMFA01010012
1.48_199.1758 [M-H]	Fatty Acyls [FA0103][α]	$C_{16}H_{26}O_2$	Hiragonic acid	https://www.lipidmaps.org/data/LMSDRecord.php?&LMID=LMFA01030137

1.52_249.1907 [M-H]	Fatty Acyls [FA0103][α]	$C_{16}H_{26}O_2$	4,7,10-hexadecatrienoic acid	https://www.lipidmaps.org/data/LMSDRecord.php?&LMID=LMFA01030134
1.98_227.2067 [M-H]	Fatty Acyls [FA0101][z]	$C_{14}H_{28}O_2$	Myristic acid	https://www.lipidmaps.org/data/LMSDRecord.php?&LMID=LMFA01010014
2.02_253.2221 [M-H]	Fatty Acyls [FA0103]	$C_{16}H_{30}O_2$	Gaidic acid	https://www.lipidmaps.org/data/LMSDRecord.php?&LMID=LMFA01030054
2.72_255.2380 [M-H]	Fatty Acyls [FA0101][z]	$C_{16}H_{32}O_2$	Palmitic acid	https://www.lipidmaps.org/data/LMSDRecord.php?&LMID=LMFA01010001
2.79_537.4898 [M-H]	Fatty Acyls [FA0701][κ]	$C_{34}H_{66}O_4$	FAHFA(16:0/5-O-18:0)	https://www.lipidmaps.org/data/LMSDRecord.php?&LMID=LMFA07011034
3.84_283.2688 [M-H]	Fatty Acyls [FA0101][z]	$C_{18}H_{36}O_2$	octadecanoic acid	https://www.lipidmaps.org/data/LMSDRecord.php?&LMID=LMFA01010018
9.20_559.4733 [M-H]	Fatty Acyls [FA0701][κ]	$C_{36}H_{64}O_4$	Mayolene-18	https://www.lipidmaps.org/data/LMSDRecord.php?&LMID=LMFA07010014

[a][PR0104]: C20 isoprenoids (diterpenes); [b][PK12]: Flavonoids; [c][ST0203]: C21 steroids (gluco/mineralocorticoids, progestogins) and derivatives; [d][PR0103]: C15 isoprenoids (sesquiterpenes); [e][PR0102]: C10 isoprenoids (monoterpenes); [f][PR0107]: C40 isoprenoids; [g][ST0202]: C19 steroids (androgens) and derivatives; [h][SP0108]: Sphingoid base analogs (tetraterpenes); [i][ST0201]: C18 steroids (estrogens) and derivatives; [j][ST0101]: Cholesterol and derivatives; [k][ST0302]: Vitamin D3 and derivatives; [L][SP00]: Other Sphingolipids; [m][GP0102]: 1-alkyl,2-acylglycerophosphocholines; [n][GP0409]: Diacylglycerophosphomonoradylglycerols; [o][GL0301]: Triacylglycerols; [p][GL0201]: Diacylglycerols; [q][GL0301]: Triacylglycerols; [r][GP0101]: Diacylglycerophosphocholines; [s][SP0301]: Ceramide phosphocholines (sphingomyelins); [t][SP0201]: N-acylsphingosines (ceramides); [u][SP0302]: Ceramide phosphoethanolamines; [v][SP0203]: N-acyl-4-hydroxysphinganines (phytoceramides); [w][SP0501]: Simple Glc series; [x][FA0301]: Prostaglandins; [y][FA0117]: Dicarboxylic acids; [z][FA0101]: Straight chain fatty acids; [α][FA0103]: Unsaturated fatty acids; [β][FA0203]: Phytoprostanes; [γ][FA0200]: Other Octadecanoids; [δ][FA0105]: Hydroxy fatty acids; [ε][FA0400]: Other Docosanoids; [ζ][FA0403]: Resolvin Ds; [η][FA0107]: Epoxy fatty acids; [κ][FA0701]: Wax monoesters.

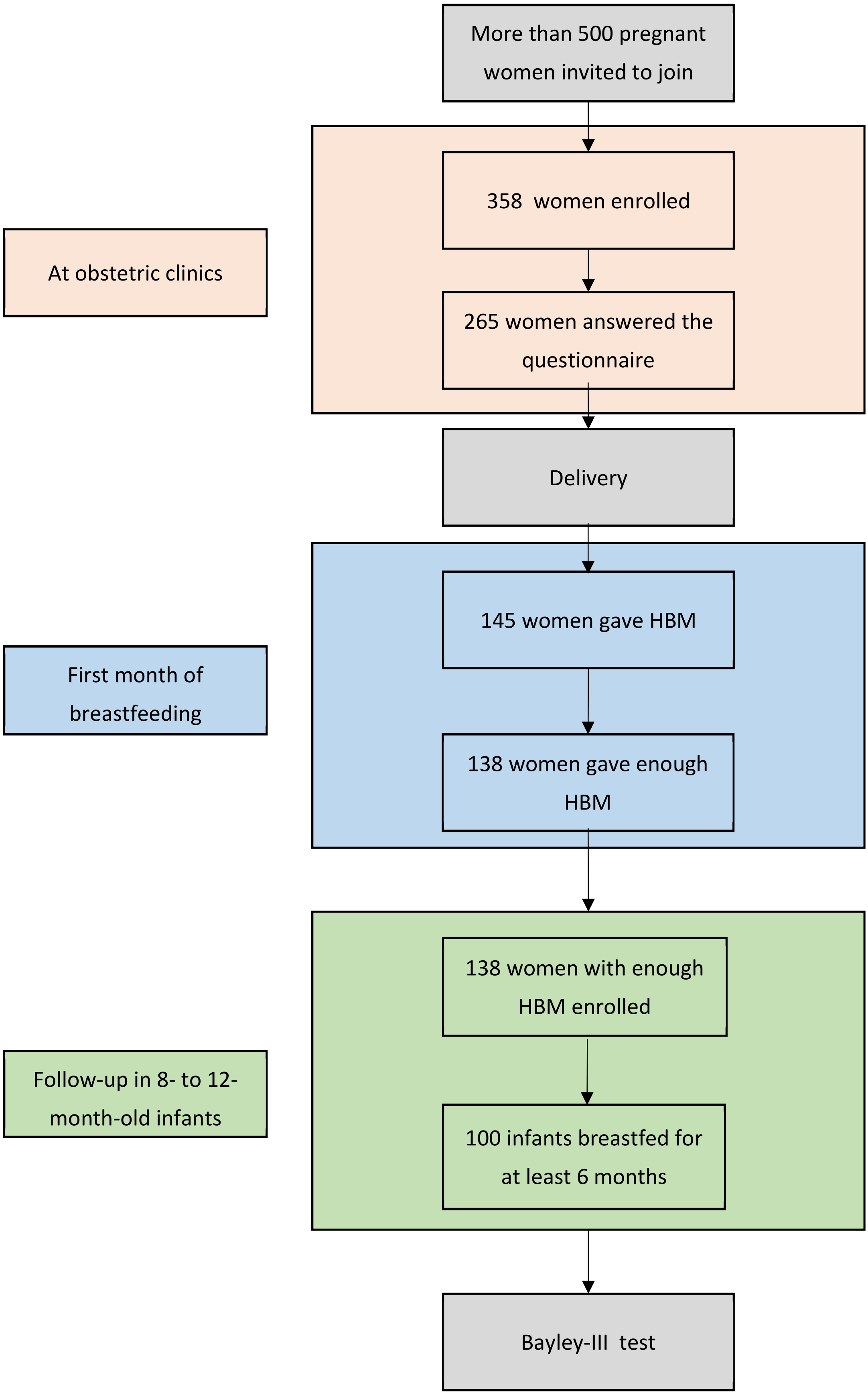

Figure S1. Flowchart showing the recruitment, enrollment, and inclusion of study participants.

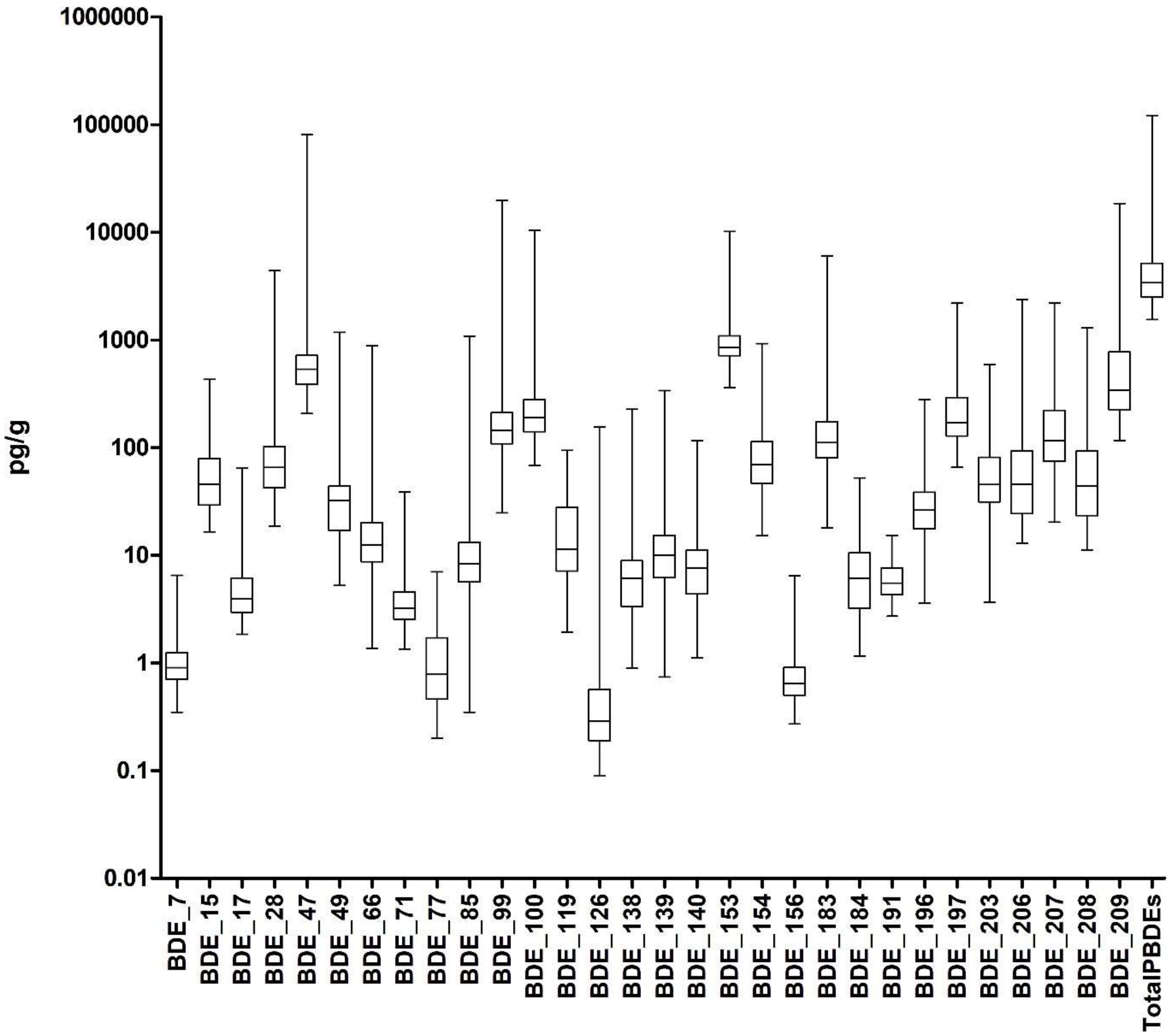

Figure S2. Box-and-whisker plots of 30 PBDE compounds and ΣPBDEs.

References

1. Siddiqi, M. A.; Laessig, R. H.; Reed, K. D., Polybrominated diphenyl ethers (PBDEs): new pollutants-old diseases. *Clin. Med. Res.* **2003,** 1, (4), 281-90.
2. Alaee, M.; Arias, P.; Sjodin, A.; Bergman, A., An overview of commercially used brominated flame retardants, their applications, their use patterns in different countries/regions and possible modes of release. *Environ. Int.* **2003,** 29, (6), 683-9.
3. Dodson, R. E.; Perovich, L. J.; Covaci, A.; Van den Eede, N.; Ionas, A. C.; Dirtu, A. C.; Brody, J. G.; Rudel, R. A., After the PBDE phase-out: a broad suite of flame retardants in repeat house dust samples from California. *Environ. Sci. Technol.* **2012,** 46, (24), 13056-66.
4. Chao, H. R.; Lin, D. Y.; Chen, K. Y.; Gou, Y. Y.; Chiou, T. H.; Lee, W. J.; Chen, S. J.; Wang, L. C., Atmospheric concentrations of persistent organic pollutants over the Pacific Ocean near southern Taiwan and the northern Philippines. *Sci. Total Environ.* **2014,** 491-492, 51-9.
5. McGrath, T. J.; Morrison, P. D.; Sandiford, C. J.; Ball, A. S.; Clarke, B. O., Widespread polybrominated diphenyl ether (PBDE) contamination of urban soils in Melbourne, Australia. *Chemosphere* **2016,** 164, 225-232.
6. Yang, Y.; Xie, Q.; Liu, X.; Wang, J., Occurrence, distribution and risk assessment of polychlorinated biphenyls and polybrominated diphenyl ethers in nine water sources. *Ecotoxicol. Environ. Saf.* **2015,** 115, 55-61.
7. Corsolini, S.; Baroni, D.; Martellini, T.; Pala, N.; Cincinelli, A., PBDEs and PCBs in terrestrial ecosystems of the Victoria Land, Antarctica. *Chemosphere* **2019,** 231, 233-239.
8. Kim, J. T.; Choi, Y. J.; Barghi, M.; Yoon, Y. J.; Kim, J. H.; Kim, J. H.; Chang, Y. S., Occurrence and distribution of old and new halogenated flame retardants in mosses and lichens from the South Shetland Islands, Antarctica. *Environ. Pollut.* **2018,** 235, 302-311.
9. Gou, Y. Y.; Que, D. E.; Chuang, C. Y.; Chao, H. R.; Shy, C. G.; Hsu, Y. C.; Lin, C. W.; Chuang, K. P.; Tsai, C. C.; Tayo, L. L., Dust levels of polybrominated diphenyl ethers (PBDEs) and polybrominated dibenzo-p-dioxins/furans (PBDD/Fs) in the Taiwanese elementary school classrooms: Assessment of the risk to school-age children. *Sci. Total Environ.* **2016,** 572, 734-741.
10. Guo, J.; Lin, K.; Deng, J.; Fu, X.; Xu, Z., Polybrominated diphenyl ethers in indoor air during waste TV recycling process. *J. Hazard. Mater.* **2015,** 283, 439-46.
11. Chen, M. W.; Castillo, B. A. A.; Lin, D. Y.; Chao, H. R.; Tayo, L. L.; Gou, Y. Y.; Chen, F. A.; Huang, K. L., Levels of PCDD/Fs, PBDEs, and PBDD/Fs in breast milk from southern Taiwan. *Bull. Environ. Contam. Toxicol.* **2018,** 100, (3), 369-375.

12. He, Y.; Peng, L.; Zhang, W.; Liu, C.; Yang, Q.; Zheng, S.; Bao, M.; Huang, Y.; Wu, K., Adipose tissue levels of polybrominated diphenyl ethers and breast cancer risk in Chinese women: A case-control study. *Environ. Res.* **2018,** 167, 160-168.

13. Lin, S. M.; Chen, F. A.; Huang, Y. F.; Hsing, L. L.; Chen, L. L.; Wu, L. S.; Liu, T. S.; Chang-Chien, G. P.; Chen, K. C.; Chao, H. R., Negative associations between PBDE levels and thyroid hormones in cord blood. *Int. J. Hyg. Environ. Health* **2011,** 214, (2), 115-20.

14. Xu, L.; Huo, X.; Zhang, Y.; Li, W.; Zhang, J.; Xu, X., Polybrominated diphenyl ethers in human placenta associated with neonatal physiological development at a typical e-waste recycling area in China. *Environ. Pollut.* **2015,** 196, 414-22.

15. Frederiksen, M.; Vorkamp, K.; Thomsen, M.; Knudsen, L. E., Human internal and external exposure to PBDEs--a review of levels and sources. *Int. J. Hyg. Environ. Health* **2009,** 212, (2), 109-34.

16. Koenig, C. M.; Lango, J.; Pessah, I. N.; Berman, R. F., Maternal transfer of BDE-47 to offspring and neurobehavioral development in C57BL/6J mice. *Neurotoxicol. Teratol.* **2012,** 34, (6), 571-80.

17. Toms, L. M.; Sjodin, A.; Harden, F.; Hobson, P.; Jones, R.; Edenfield, E.; Mueller, J. F., Serum polybrominated diphenyl ether (PBDE) levels are higher in children (2-5 years of age) than in infants and adults. *Environ. Health Perspect.* **2009,** 117, (9), 1461-5.

18. Chao, H. R.; Tsou, T. C.; Huang, H. L.; Chang-Chien, G. P., Levels of breast milk PBDEs from southern Taiwan and their potential impact on neurodevelopment. *Pediatr. Res.* **2011,** 70, (6), 596-600.

19. Gascon, M.; Fort, M.; Martinez, D.; Carsin, A. E.; Forns, J.; Grimalt, J. O.; Santa Marina, L.; Lertxundi, N.; Sunyer, J.; Vrijheid, M., Polybrominated diphenyl ethers (PBDEs) in breast milk and neuropsychological development in infants. *Environ. Health Perspect.* **2012,** 120, (12), 1760-5.

20. Shy, C. G.; Huang, H. L.; Chang-Chien, G. P.; Chao, H. R.; Tsou, T. C., Neurodevelopment of infants with prenatal exposure to polybrominated diphenyl ethers. *Bull. Environ. Contam. Toxicol.* **2011,** 87, (6), 643-8.

21. Braun, J. M.; Yolton, K.; Stacy, S. L.; Erar, B.; Papandonatos, G. D.; Bellinger, D. C.; Lanphear, B. P.; Chen, A., Prenatal environmental chemical exposures and longitudinal patterns of child neurobehavior. *Neurotoxicology* **2017,** 62, 192-199.

22. Herbstman, J. B.; Sjodin, A.; Kurzon, M.; Lederman, S. A.; Jones, R. S.; Rauh, V.; Needham, L. L.; Tang, D.; Niedzwiecki, M.; Wang, R. Y.; Perera, F., Prenatal exposure to PBDEs and neurodevelopment. *Environ. Health Perspect.* **2010,** 118, (5), 712-9.

23. Adgent, M. A.; Hoffman, K.; Goldman, B. D.; Sjodin, A.; Daniels, J. L., Brominated flame retardants in breast milk and behavioural and cognitive development at 36 months. *Paediatr. Perinat. Epidemiol.* **2014,** 28, (1), 48-57.

24. Hertz-Picciotto, I.; Bergman, A.; Fangstrom, B.; Rose, M.; Krakowiak, P.; Pessah, I.; Hansen, R.; Bennett, D. H., Polybrominated diphenyl ethers in relation to autism and developmental delay: a case-control study. *Environ Health* **2011,** 10, (1), 1.

25. Kao, C. C.; Que, D. E.; Bongo, S. J.; Tayo, L. L.; Lin, Y. H.; Lin, C. W.; Lin, S. L.; Gou, Y. Y.; Hsu, W. L.; Shy, C. G.; Huang, K. L.; Tsai, M. H.; Chao, H. R., Residue levels of organochlorine pesticides in breast milk and its associations with cord blood thyroid hormones and the offspring's neurodevelopment. *Int. J. Environ. Res. Public Health* **2019,** 16, (8).

26. Shy, C. G.; Huang, H. L.; Chao, H. R.; Chang-Chien, G. P., Cord blood levels of thyroid hormones and IGF-1 weakly correlate with breast milk levels of PBDEs in Taiwan. *Int. J. Hyg. Environ. Health* **2012,** 215, (3), 345-51.

27. Lowe, J. R.; Erickson, S. J.; Schrader, R.; Duncan, A. F., Comparison of the Bayley II Mental Developmental Index and the Bayley III Cognitive Scale: are we measuring the same thing? *Acta Paediatr.* **2012,** 101, (2), e55-8.
